# 3D-bioprinting of patient-derived cardiac tissue models for studying congenital heart disease

**DOI:** 10.3389/fcvm.2023.1162731

**Published:** 2023-05-24

**Authors:** Jayne T. Wolfe, Wei He, Min-Su Kim, Huan-Ling Liang, Akankshya Shradhanjali, Hilda Jurkiewicz, Bonnie P. Freudinger, Andrew S. Greene, John F. LaDisa, Lobat Tayebi, Michael E. Mitchell, Aoy Tomita-Mitchell, Brandon J. Tefft

**Affiliations:** ^1^ Department of Biomedical Engineering, Medical College of Wisconsin & Marquette University, Milwaukee, WI, United States; ^2^ Department of Surgery, Medical College of Wisconsin, Milwaukee, WI, United States; ^3^ Engineering Core, Medical College of Wisconsin, Milwaukee, WI, United States; ^4^ The Jackson Laboratory, Bar Harbor, ME, United States; ^5^ Department of Pediatrics - Section of Cardiology, Children’s Wisconsin, Milwaukee, WI, United States; ^6^ The Herma Heart Institute, Children’s Wisconsin, Milwaukee, WI, United States; ^7^ Cardiovascular Center, Medical College of Wisconsin, Milwaukee, WI, United States; ^8^ School of Dentistry, Marquette University, Milwaukee, WI, United States

**Keywords:** 3D-bioprinting, hydrogel, induced pluripotent stem cell, cardiomyocyte, congenital heart disease, hypoplastic left heart syndrome

## Abstract

**Introduction:**

Congenital heart disease is the leading cause of death related to birth defects and affects 1 out of every 100 live births. Induced pluripotent stem cell technology has allowed for patient-derived cardiomyocytes to be studied in vitro. An approach to bioengineer these cells into a physiologically accurate cardiac tissue model is needed in order to study the disease and evaluate potential treatment strategies.

**Methods:**

To accomplish this, we have developed a protocol to 3D-bioprint cardiac tissue constructs comprised of patient-derived cardiomyocytes within a hydrogel bioink based on laminin-521.

**Results:**

Cardiomyocytes remained viable and demonstrated appropriate phenotype and function including spontaneous contraction. Contraction remained consistent during 30 days of culture based on displacement measurements. Furthermore, tissue constructs demonstrated progressive maturation based on sarcomere structure and gene expression analysis. Gene expression analysis also revealed enhanced maturation in 3D constructs compared to 2D cell culture.

**Discussion:**

This combination of patient-derived cardiomyocytes and 3D-bioprinting represents a promising platform for studying congenital heart disease and evaluating individualized treatment strategies.

## 1. Introduction

Congenital heart disease (CHD) occurs in approximately 1 in 100 live births (1). Hypoplastic left heart syndrome (HLHS) is a clinically severe form of CHD characterized by atresia/stenosis of the aortic and mitral valves, and hypoplasia of the left ventricle and aorta. HLHS is responsible for significant mortality in pediatric CHD and affects more than 1 in every 4,000 live births (2). HLHS is the most common cause of premature death among all congenital malformations. The etiology of HLHS, which is understood in only a small percentage (∼10%) of cases, consists mainly of single gene defects and chromosomal abnormalities, plus environmental factors in a minority of instances. The unknown etiology of the great majority (∼90%) of human HLHS derives largely from an absence of suitable animal models, and the inability to experiment on the human heart during embryonic and fetal stages of development. However, the recent breakthrough enabling somatic cells from patients to be re-programmed to the pluripotent state—i.e., to induced pluripotent stem cells (iPSCs)—now provides the ability to monitor the development of selected patient-matched cell types as they differentiate *in vitro*.

We have differentiated these iPSCs into cardiomyocytes (iPSC-CMs), which constitute an invaluable *in vitro* model system for studying CHD and evaluating potential treatments (3). While valuable insights from studying iPSC-CMs in 2D culture have been gained, it is well established that 3D cell culture environments provide a more accurate microenvironment that better recapitulates cell behaviour *in vitro* (4). For example, 3D cell culture has been shown to more accurately reproduce gene expression (5), drug efficacy and toxicity (6), drug metabolism (7), and drug sensitivity (8). Most relevant to the proposed study, 3D cell culture of cardiomyocytes has been shown to improve maturity and retention of contractile phenotype (9, 10).

Several recent studies have employed the bioengineering approach of 3D-bioprinting to create a 3D tissue construct seeded with CMs (11–13). In order to establish a more physiologically accurate model of congenital heart disease, the goal of this study is to use a 3D-bioprinting approach to create a 3D culture environment for our patient-specific iPSC-CMs. The 3D-bioprinting approach allows for highly controlled and repeatable fabrication conditions. 3D-bioprinting also allows for tailoring the microenvironment by altering the extracellular matrix (ECM) components and growth factors within the hydrogel biomaterial. Cells are then supported within a 3D network of crosslinked ECM that guides cell behaviour and tissue maturation similar to physiologic conditions.

3D-bioprinting allows for several parameters to be optimized in order to achieve desired outcomes. These include construct geometry, print pattern, layer thickness, temperature, extrusion pressure, print speed, and nozzle diameter. The most critical parameters are bioink composition and crosslinking strategy, and we found our iPSC-CMs to be especially sensitive to these. We explored a variety of hydrogel bioinks, including one based on laminin-521. Laminins are the most abundant non-collagenous proteins in the basement membrane, the cell-adherent ECM that stabilizes parenchymal architecture. The laminins not only mediate basement membrane self-assembly during development (14, 15), but are vital and crucial to processes of cellular recognition, adhesion, migration, and proliferation. Each laminin is a heterotrimeric protein, containing three chains each. Each subunit is encoded by its own gene: LAMA(α), LAMB(β), and LAMC(γ) (16). The alpha subunits mediate basement membrane self-assembly and cell-cell adhesion, via binding to integrins in the cell membrane through its LG domains in the alpha subunit's C-terminus (15, 17). Laminin-integrin binding has been shown to regulate cell proliferation and migration (14). During embryonic development, CMs and endothelial cells (ECs) express laminin α-4 and laminin α-5 as a component of laminins 8(α4β1γ1), 9(α4β2γ1) and 14(α4β2γ4), and laminin α-2 and laminin α-5 chains at adult stages. Laminin-521 is expressed and secreted by iPSCs in the inner cell mass of the embryo.

Here, we describe a repeatable method to quantitatively assess iPSC-CM sarcomere quality and maturation in a 3D culture environment using 3D-bioprinting with a commercially available bioink, GelXA Laminink-521. We show healthy sarcomeres develop and mature appropriately over time within the 3D-bioprinted cardiac tissue constructs. The goal of the study is to develop and validate a straightforward methodology that allows researchers from a variety of disciplines to use 3D-bioprinting to study iPSC-CMs in a physiologically relevant tissue model.

## 2. Materials and methods

### 2.1. Generation of cardiomyocytes from induced pluripotent stem cells

Skin biopsies were collected from patients with CHD and healthy family members and stored in a tissue bank (18). Dermal fibroblasts from these biopsies were isolated and reprogrammed into iPSCs using Sendai reprogramming as previously described (18). iPSCs were then differentiated into iPSC-CMs using our established protocol (19). Briefly, CHIR and a low level of Activin-A at early stage and IWP2 at later stage are used to induce cardiomyocyte differentiation. Beginning at differentiation day 15, contracting CMs are cultured in RPMI/B-27 medium (ThermoFisher Scientific, Waltham MA) supplemented with 20% fetal bovine serum (FBS) (ThermoFisher Scientific, Waltham MA). This protocol yields rhythmically contracting CMs around differentiation day 6.

For this study, iPSC-CMs derived from a healthy female family member of a patient with HLHS were used. This family harbours a variant in the MYH6 gene which manifests in the head domain of α-myosin heavy chain (α-MHC) and adversely affects sarcomere structure and function (3, 18). This will allow for MYH6 variant-carrying cells to be 3D-bioprinted and compared in future studies.

### 2.2. 3D-bioprinting and crosslinking

At differentiation day 15 ± 1, iPSC-CMs were 3D-bioprinted into nascent tissue constructs. Approximately 5.0 × 10^7^–1.5 × 10^8^ cells were washed with phosphate buffered saline (PBS) (ThermoFisher Scientific, Waltham MA) and lifted from the culture dish using 0.25% trypsin solution (ThermoFisher Scientific, Waltham MA) for 10 min at 37°C. The cell suspension was then centrifuged at 1,000 rpm for 5 min and the cell pellet was resuspended in 100 μL of culture medium supplemented with 1 μL of 5 mM Y-27632 Rho Kinase (ROCK) inhibitor (SelleckChem, Houston TX). The cell suspension was mixed with 0.9 ml of 37°C GelXA Laminink-521 bioink (CellInk, Boston MA) to a final volume of 1.0 ml. The cell/bioink mixture was then loaded into a 3 ml syringe (CellInk, Boston MA) and centrifuged at 1,000 rpm for 5 min to remove air bubbles and kept at 37°C.

An Inkredible + bioprinter (CellInk, Boston MA) was used for 3D-bioprinting. The syringe was loaded into the bioprinter and cooled to 25°C. A 22 G nozzle (CellInk, Boston MA) was attached to the syringe for printing. A pressure of 18–30 kPa was applied as appropriate and constructs were printed in 3 vertical layers to dimensions of 4.0 mm × 4.0 mm × 0.6 mm (LxWxH). Creo Parametric 3D Modeling software (PTC, Boston MA) was used to generate the geometry and the model was imported into HeartWare software (CellInk, Boston MA) to convert into G-code commands for bioprinting (Supplementary File S1). Constructs were then crosslinked by 15 s of exposure to 365 nm ultraviolet light followed by 1 min exposure to a 50 mM CaCl_2_ crosslinking solution (CellInk, Boston MA).

### 2.3. 3D tissue culture

Following crosslinking, constructs were washed in PBS and cultured in RPMI/B-27 medium supplemented with 20% FBS and 1% antibiotic-antimycotic (ThermoFisher Scientific, Waltham MA). Cultures were incubated at 37°C, 5% CO_2_, and 100% humidity. Culture medium was exchanged every 3 days.

### 2.4. Video analysis for contraction dynamics

Samples were observed and imaged using a light microscope on days 5, 10, 15, 20, 25, and 30 after printing. Videos were recorded at 100x magnification, 2,880 × 2,600 pixels, and 5 frames per second.

Contraction dynamics were calculated from the videos using a MATLAB program by Shradhanjali et al. (20). The program uses the adaptive reference—digital image correlation (AR-DIC) method to calculate the maximum displacement of each pixel on the construct during the contraction cycle. The maximum displacements of each pixel are then averaged to obtain the maximal averaged displacement for the sample.

### 2.5. Immunostaining for molecular structure

3D-bioprinted constructs were selected for immunostaining on days 5, 10, 20, and 30 after printing. Samples were rinsed three times in PBS and fixed in 4% paraformaldehyde solution (Electron Microscopy Sciences, Hatfield PA) for 15 min at room temperature under agitation. Samples were rinsed three times in PBS and permeabilized in 0.2% Triton X-100 solution (Sigma-Aldrich, St. Louis MO) in PBS for 15 min at room temperature under agitation. Permeabilization solution was removed and samples were labelled with primary antibodies against GATA-4 (36966S, Cell Signaling Technology, Danvers MA) at 1:400, α-actinin (ab9465, Abcam, Boston MA) at 1:250, and TNNT2 (MA5-12960, ThermoFisher Scientific, Waltham MA) at 1:300 in PBS overnight at 4°C under agitation. Samples were rinsed five times in PBS and labelled with the secondary antibodies donkey anti-rabbit Alexa Fluor 594 (A-21207, ThermoFisher Scientific, Waltham MA) at 1:750 and goat anti-mouse Alexa Fluor 488 (A-21121, ThermoFisher Scientific, Waltham MA) at 1:750 in PBS for 1 h at room temperature under agitation. Samples were rinsed with PBS and mounted on slides with ProLong Gold Antifade Mountant (ThermoFisher Scientific, Waltham MA). Samples were imaged at random fields under 630x magnification using a confocal microscope (Zeiss, Jena, Germany).

Sarcomere length was measured as the average distance between consecutive z-discs based on α-actinin staining. This was accomplished using the rectangle tool in ZEN microscopy software (Zeiss, Jena, Germany) to create an intensity plot along the length of the myofibrils. The average distance between intensity peaks represents the sarcomere length. For each bioprinted sample, sarcomeres within *n* = 10–15 myofibrils from cells in random locations were measured to determine the average sarcomere length for the sample.

Sarcomere organization index was measured using a MATLAB program by Hinson et al. (21) as described elsewhere (22, 23). Briefly, the α-actinin channel is converted to binary and gridded. Intensity plots are created along each myofibril. A Fast Fourier Transform (FFT) is used to create power spectra, which are then normalized to give an organization index of 0–1.

### 2.6. RT-qPCR for gene expression

3D-bioprinted constructs were selected for gene expression analysis on days 1, 5, 10, 20, and 30 after printing. For comparison, 2D cultured iPSC-CMs at differentiation day 24 (equivalent timepoint to constructs 10 days after 3D-bioprinting) were included as well. Samples were rinsed with PBS and RNA was extracted using the RNeasy Plus Micro Kit (Qiagen, Hilden, Germany). Starting with 47–272 ng of total RNA per sample, QuantiTect Reverse Transcription Kit (Qiagen, Hilden, Germany) was used to synthesize a cDNA library. Briefly, a gDNA elimination reaction was performed with gDNA wipeout buffer at 42°C for 2 min. Then the RNA was added to the reverse transcription master mix at 42°C for 30 min to synthesize cDNA. The reaction was then stopped by exposure to 95°C for 3 min.

RT-qPCR was performed using QuantStudio 7 Real-Time PCR system (ThermoFisher Scientific, Waltham MA) in triplicates for each sample with TaqMan assay (ThermoFisher Scientific, Waltham MA) using AptaTaq Genotyping master mix (Roche Diagnostics, Mannheim, Germany). It was done in duplex with the housekeeping gene glyceraldehyde-3-phosphate dehydrogenase (GAPDH) serving as endogenous control. RT-qPCR was performed with initial 50°C for 2 min, 95°C for 10 s, then 40 cycles of 95°C for 15 s and 60°C for 1 min. Target genes were alpha actin skeletal muscle (ACTA1), myosin light chain 2 (MYL2), sarcolipin (SLN), bridging integrator 1 (BIN1), junctophilin 2 (JPH2), potassium inwardly rectifying channel subfamily J member 4 (KCNJ4), myosin heavy chain 6 (MYH6), myosin heavy chain 7 (MYH7), myosin light chain 7 (MYL7), and troponin I, cardiac muscle (TNNI3). Relative gene expression levels were calculated using the delta delta CT method.

### 2.7. Statistical analysis

Differences between means were compared using a *t*-test for two groups and ANOVA with Tukey's multiple comparisons test for three or more groups. Differences were considered statistically significant for *p* < 0.05.

## 3. Results

### 3.1. Optimization of 3D-bioprinting protocol

Initial 3D-bioprinting attempts resulted in tissue constructs with poor shape fidelity and mechanical integrity, low cell viability, and/or undetectable contraction based on visual inspection (data not shown). CMs were not viable when printed using a bioink consisting of 7% gelatin (Sigma, St. Louis MO), 3% alginate (Sigma, St. Louis MO), and 10 μg/ml type I collagen (Corning, Corning NY) and crosslinked using 100 mM CaCl_2_ solution (Sigma, St. Louis MO). CMs were viable and sparsely contractile when printed using Laminink-521 bioink (CellInk, Boston MA), which consists of alginate, cellulose, and a combination of laminins including laminin-521. CMs were viable and uniformly contractile when printed using GelXA Laminink-521 bioink, which consists of gelatin, alginate, xanthum gum, and a combination of laminins including laminin-521 (Supplementary Videos S1–S2).

Laminink-521 must be crosslinked by CaCl_2_ solution whereas GelXA Laminink-521 can crosslink by either ultraviolet (UV) irradiation or CaCl_2_ solution. UV irradiation can be damaging to all cell types and CMs are sensitive to calcium ions. We therefore determined that crosslinking GelXA Laminink-521 using a partial dose of UV (15 s exposure) combined with a partial dose of CaCl_2_ (50 mM solution for 1 min) achieved a superior balance between cell viability, cell function, and construct integrity compared to a full dose of UV or a full dose of CaCl_2_.

**Figure 1:**
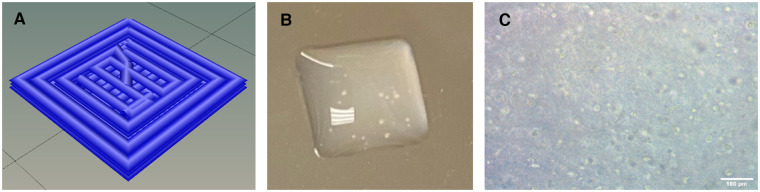
(**A**) Schematic of geometrical design showing the print paths. (**B**) Macroscopic photograph of 3D-bioprinted construct. 4 mm x 4 mm x 0.6 mm. (**C**) Microscopy image of 3D-bioprinted construct showing cellular content. Scalebar 100 µm.

Furthermore, we determined a cell density of approximately 5.0 × 10^7^–1.0 × 10^8^ cells/ml was necessary to achieve contraction. Cell densities lower than this failed to achieve the intercellular communication necessary for coordinated contraction across the entire construct. The high cell density requirement limited the size of the constructs we could print in this study to 4.0 mm × 4.0 mm × 0.6 mm, or 9.6 μL. The height of 0.6 mm was chosen because it corresponded to 3 layers of bioink and was consistently printable and crosslinkable (Figure 1).

### 3.2. Immunostaining

**Figure 2:**
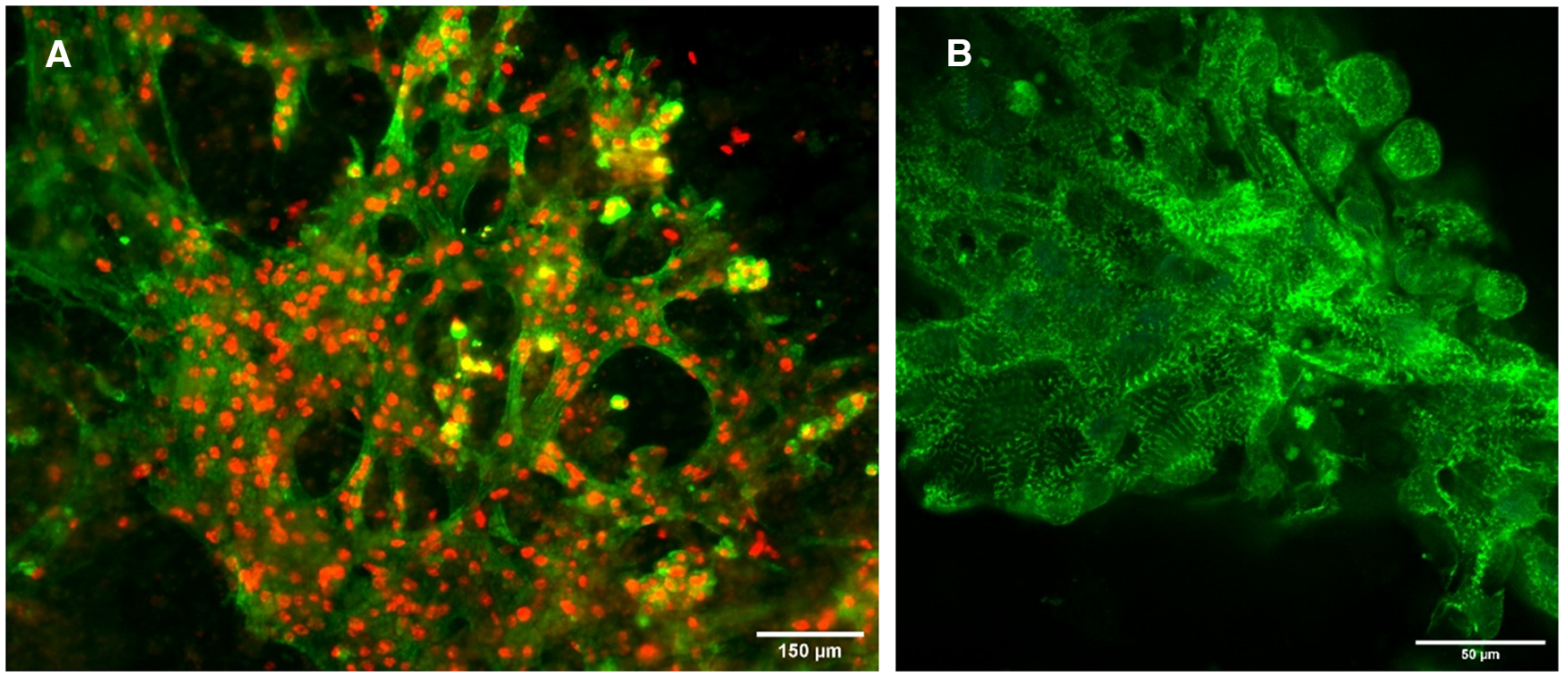
Immunofluorescence images of constructs. (**A**) Constructs with wildtype cells stained for TNNT2 (green) and GATA4 (red). Scalebar 150 µm. (**B**) Constructs with wildtype cells stained for α-actinin (green). Scalebar 50 µm.

Immunostaining of the 3D-bioprinted constructs revealed intact CM phenotype. Cells uniformly stained positive for the CM markers TNNT2 and GATA4 in the cytoplasm and nuclei, respectively (Figure 2). Typical sarcomere structure was also observed as visualized by α-actinin staining in constructs with wildtype cells (Figure 2). The sarcomeres appeared to be mature and organized.

### 3.3. Sarcomere length

**Figure 3:**
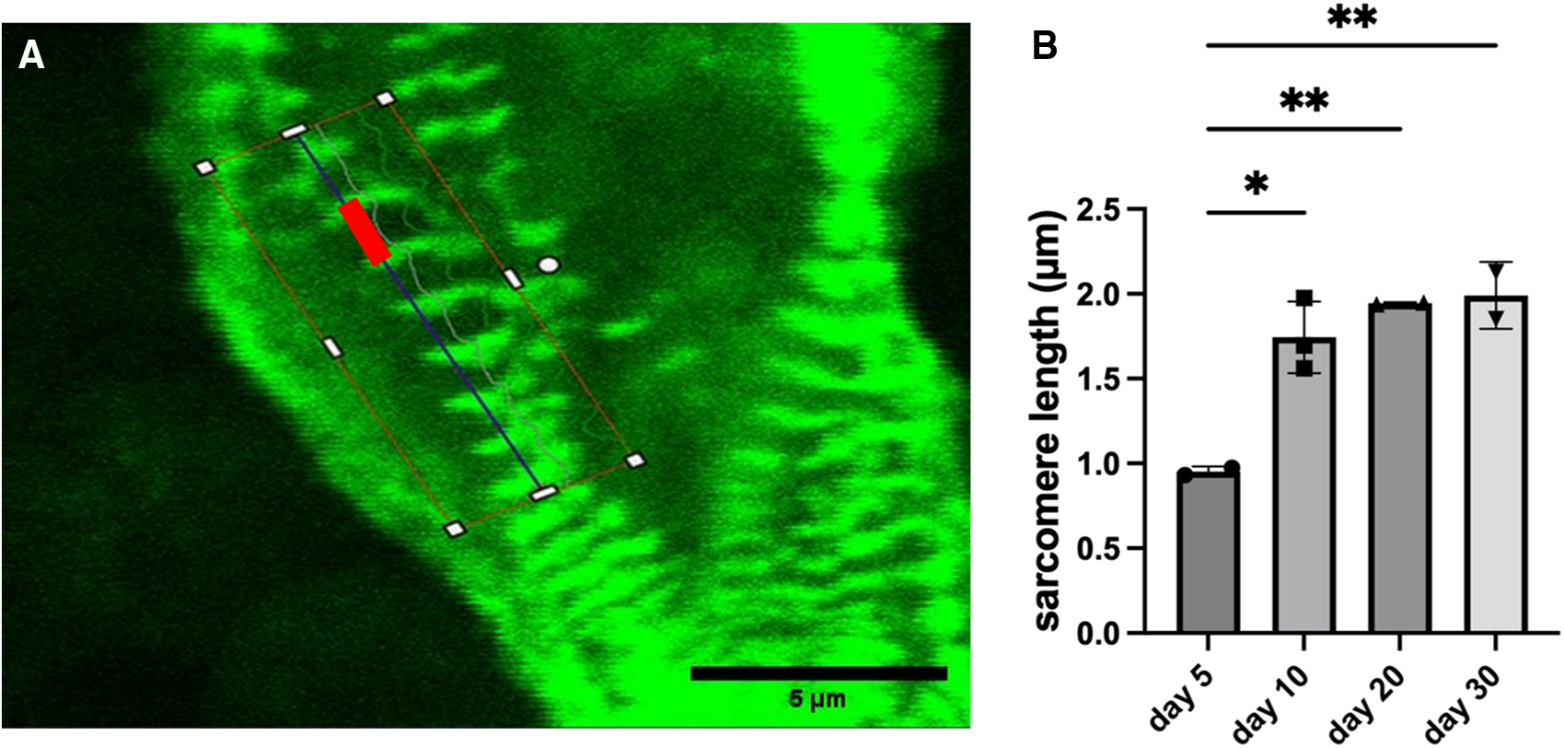
Measurements of sarcomere length for constructs with wildtype cells. (**A**) Example image showing a typical measurement of sarcomere length (red bar). Scalebar 5 µm. (**B**) Sarcomere length with data shown as mean ± standard deviation. *n* = 2–3. *p < 0.05.

Sarcomere length progressively increased for constructs with wildtype cells. The length started at approximately 0.95 μm on day 5 after bioprinting and reached approximately 1.99 μm on day 30 (Figure 3). Sarcomeres lengthen as they mature, so this indicates a steady maturation of the sarcomere structures. The sarcomere length stabilized by day 30 indicating the sarcomeres had finished maturing.

### 3.4. Sarcomere organization index

**Figure 4:**
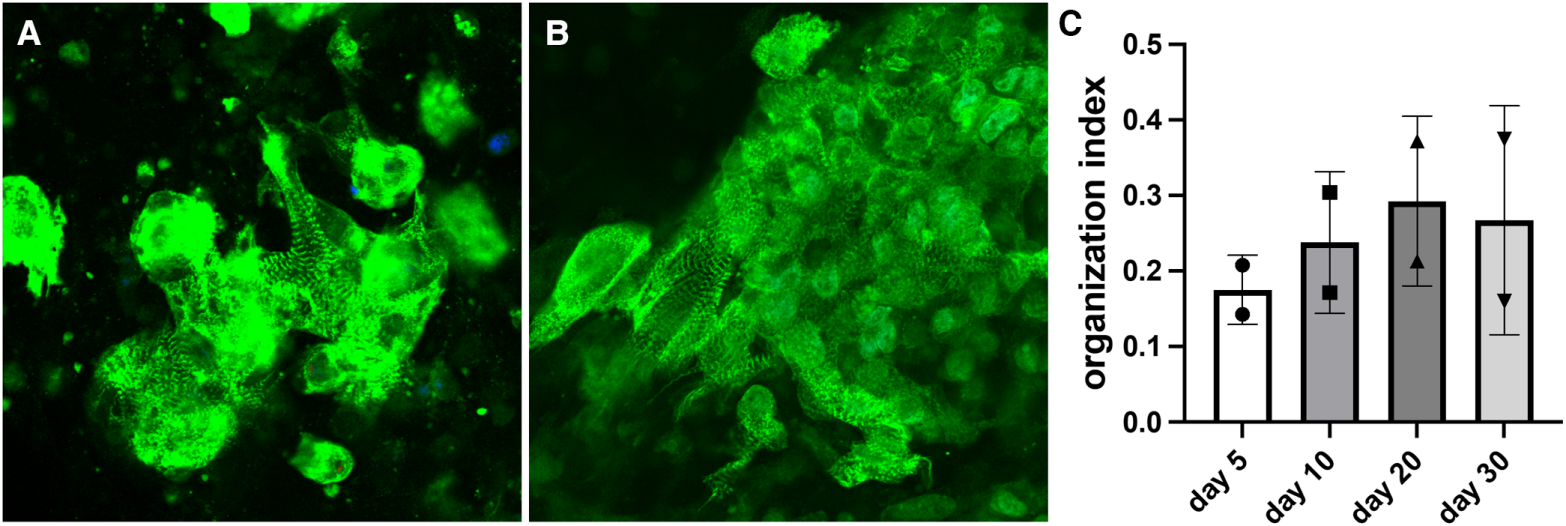
Measurements of organization index for constructs with wildtype cells. (**A**) Representative image of the day 5 bioprinted construct (organization index = 0.151). (**B**) Representative image of the day 30 bioprinted construct (organization index = 0.276). (**C**) Organization index data shown as mean ± standard deviation. *n* = 2.

Organization index demonstrated a similar pattern as sarcomere length. Organization index progressively increased for constructs with wildtype cells. The value started at approximately 0.18 on day 5 after bioprinting and reached approximately 0.27 on day 30 (Figure 4). Sarcomeres become more organized as they mature, so this indicates a steady maturation of the sarcomere structures. The organization index stabilized by day 30 indicating the sarcomeres had finished maturing.

### 3.5. Contraction dynamics

**Figure 5:**
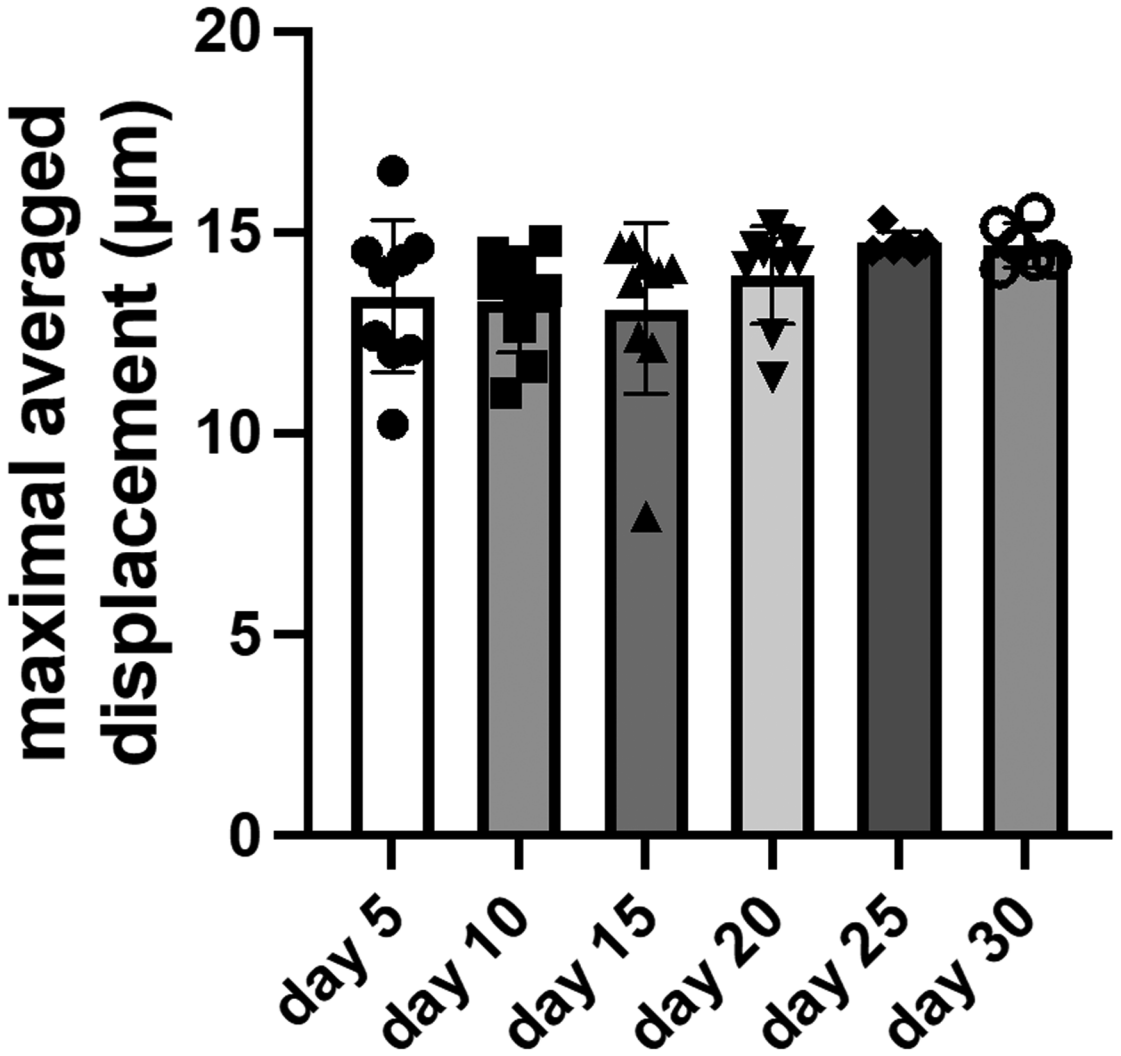
Measurements of maximal averaged displacement for constructs with wildtype cells. Data shown as mean ± standard deviation. *n* = 6–9.

The maximal averaged displacement during contraction was consistent across all time points for constructs with wildtype cells (Figure 5). The displacement was 13.4 μm on day 5 and only slightly increased to 14.7 μm on day 30. The displacement remained within ±10% of the day 5 baseline value. This indicates the constructs quickly achieved full contractility after 3D-bioprinting and maintained full contractility for several weeks in culture.

### 3.6. Gene expression

**Figure 6:**
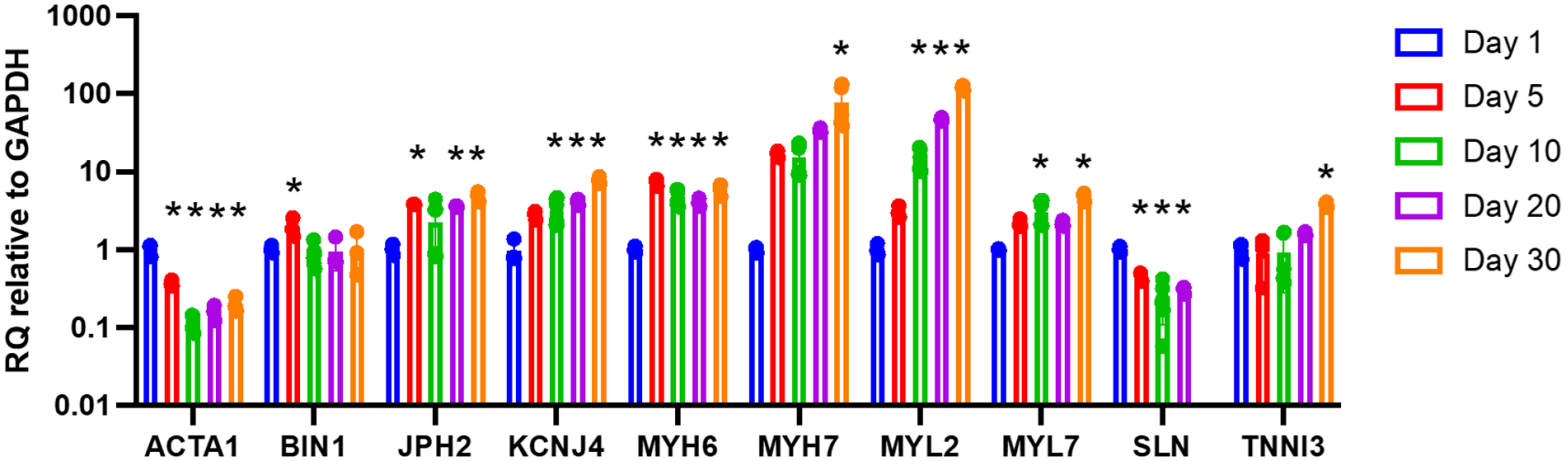
Longitudinal gene expression for 3D-bioprinted constructs with wildtype cells. Data shown as expression relative to day 1 baseline. Data shown as mean ± standard deviation. *n* = 2–6. * *p* < 0.05 compared to day 1 baseline.

Gene expression analysis was performed for *ACTA1*, *MYL2*, *SLN*, *BIN1*, *JPH2*, *KCNJ4*, *MYH6*, *MYH7*, *MYL7*, and *TNNI3* by RT-qPCR. Longitudinal analysis of constructs with wildtype cells demonstrates a progressive increase in the expression of *MYL2*, *KCNJ4*, *MYH7*, *MYL7*, and *TNNI3* until reaching 119.0×, 8.2×, 67.5×, 4.7×, and 4.0×, respectively, on day 30 compared to day 1 baseline (Figure 6). *JPH2* and *MYH6* both initially increased then levelled before reaching approximately 4.8× and 5.8×, respectively, on day 30 compared to day 1 baseline. *ACTA1* and *SLN* both initially decreased then levelled before reaching approximately 0.20× on day 30 and 0.30× on day 25, respectively, compared to day 1 baseline. *BIN1* initially increased to 1.9× on day 5 compared to day 1 baseline before returning to baseline.

**Figure 7:**
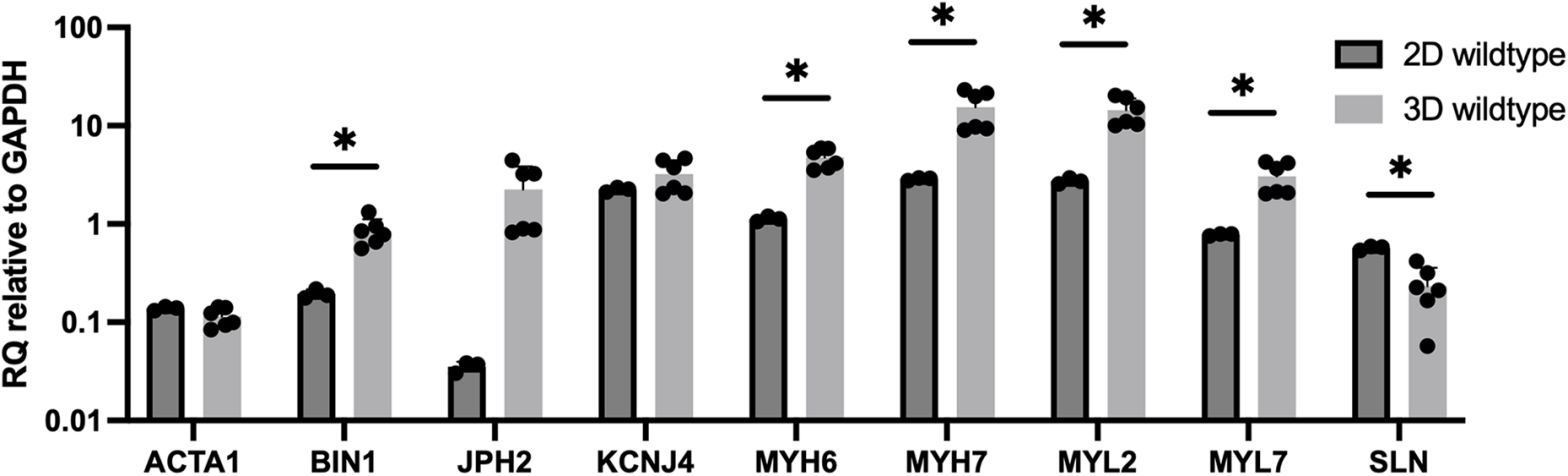
Gene expression for 3D-bioprinted constructs with wildtype cells (25 days after differentiation and 10 days after 3D-bioprinting) vs. 2D culture of wildtype cells (24 days after differentiation). Data shown as expression relative to 3D wildtype day 1 baseline. Data shown as mean ± standard deviation. *n* = 3–6. *p < 0.05.

Gene expression for 3D-bioprinted constructs with wildtype cells compared to 2D culture of wildtype cells demonstrates similar expression for *ACTA1* (0.11× vs. 0.14× compared to day 1 baseline) (Figure 7). *MYL2*, *BIN1*, *JPH2*, *MYH6*, *MYH7*, and *MYL7* expression are higher in 3D-bioprinted constructs compared to 2D culture (13.8× vs. 2.7×, 0.82× vs. 0.19×, 1.8× vs. 0.04×, 4.7× vs. 1.1×, 14.2× vs. 2.9×, and 2.9× vs. 0.78×, respectively, compared to day 1 baseline). *KCNJ4* expression is slightly higher in 3D-bioprinted constructs compared to 2D culture (3.2× vs. 2.4× compared to day 1 baseline). *SLN* expression is lower in 3D-bioprinted constructs compared to 2D culture (0.20× vs. 0.57× compared to day 1 baseline).

## 4. Discussion

The molecular mechanisms controlling HLHS and cardiomyopathy, including the role of sarcomere genes such as *MHY6*, remains to be elucidated. Further, any potential therapies developed for HLHS need to be tested in an appropriate model before moving into humans. The goal of this work was to establish a living cardiac tissue model derived from patient-specific iPSC-CMs to better understand the role of genetic variants contributing to CHD in a relevant 3D environment. We aimed to develop this model for testing potential therapeutics and developing bioengineered tissues that may aid in reconstructive cardiac surgery in the future.

The ability to derive patient-specific iPSC-CMs using somatic cells makes them a favourable cell type for studying CHD, especially those with rare genetic variants (24). However, the relatively immature phenotype exhibited by iPSC-CMs compared to human adult CMs remains a significant limitation of their use (9). Therefore, we developed 3D constructs to better mimic native tissue and encourage maturation of the CMs to exhibit more features of the native adult CM. Additionally, it is known that the behaviour of CMs differs vastly from 2D to 3D culture. In native tissue, cells are embedded in an ECM with stimuli that causes appropriate gene expression responses (4). Bioprinting with an ECM-based bioink allowed us to create 3D tissue constructs, which offer a more relevant microenvironment to study genetic variants in sarcomere proteins and better recapitulate disease phenotypes than in 2D culture.

One of the major benefits of 3D-bioprinting is the ability to fabricate complicated geometries. This is not an important capability for cardiac tissue engineering because engineered cardiac tissue can be easily trimmed to the appropriate dimensions during surgical implantation. For this reason, we bioprinted a simple rectangular prism shape in this study. The rationale for using 3D-bioprinting in this study is derived from another major benefit which is the ability to micropattern multiple cell types within a printed structure in a precise and controlled manner (25). Recent studies have highlighted the importance of co-culture for accurate CM behaviour (26–30). Future studies will explore co-culture patterning of cells such as cardiac fibroblasts, endothelial cells, and mesenchymal stem cells to bioengineer physiologically accurate cardiac tissue with perfusable vascular networks. Our present work focused on optimizing the bioprinting conditions which would allow the iPSC-CMs to survive the printing process and remain viable up to 30 days post-printing. GelXA Laminink-521 is a commercially available bioink that was explored due to the high laminin protein content, which supports growth of many cell types including CMs. Laminin-521 has also been shown to be a critical enhancer of CM maturation (31).

A cross-linking method is needed to form a solid hydrogel scaffold after extrusion from the syringe. We determined a combination of UV light and chemical cross-linking with CaCl_2_ was the best option for maintaining the iPSC-CMs in the printed shape without adversely affecting cell viability. The GelXA Laminink-521 bioink is curable with both UV and CaCl_2_. A previous study determined UV exposure time of 45 s caused no significant effect on cell viability over a week after exposure (32). In this study, we used 15 s of UV exposure to stay well within the safe range. We combined this with CaCl_2_, which is an ionic crosslinker that causes rapid gelation of the alginate component of the bioink, to further strengthen the hydrogel. A simple square geometry was chosen for this study so we could establish ideal conditions for bioprinting and cell culture. Through multiple iterations of layer patterns, layer numbers, overall dimensions, and cell density, we determined an optimal cell density and structure that consistently yielded constructs with uniformly beating CMs.

Through visualization of the cells with light microscopy, beating CMs were evident in our 3D-bioprinted constructs as soon as 3 days after bioprinting. The cells began with a more rounded appearance in the constructs, displaying regions of beating. Over time, the cells elongated and caused coordinated contraction of the whole tissue construct approximately 1 week after printing. Video analysis allowed us to interpret the maximum averaged displacement for the beating constructs, providing insight into their overall contractility.

Sarcomere length and organization were analysed as a means of determining CM maturation and alignment. It is known that an adult human CM has a sarcomere length of approximately 2.2 μm in the relaxed state (33). However, immature stem cell derived CMs tend to have a sarcomere length closer to 1.65 μm (34). Our analysis showed the average sarcomere length of cells in the bioprinted constructs started at approximately 1 μm and increased to approximately 2 μm by day 30. This aligns with similar results from other groups investigating maturation of stem cell derived CMs (34). In addition to growing longer, the increasing trend in organization index indicates the sarcomeres also become more organized during maturation in the 3D environment with laminin-521. Other groups have also observed an increase in sarcomere organization of iPSC-CMs cultured in ECM (35).

Gene expression data using a panel of genes important in CM maturation and function also supports improved maturation of the iPSC-CMs in the bioengineered 3D constructs compared to 2D culture. *ACTA1* encodes skeletal muscle alpha-actin, which importantly participates in the movement and contraction of CMs. *ACTA1* tends to be highly expressed early in human embryonic development but declines to negligible levels by birth (36). In the bioprinted constructs, high *ACTA1* levels were observed on day 1 after bioprinting but lowered by day 30 to match levels of 2D controls. We suspect *ACTA1* expression is high immediately after printing due to the stress from the printing process, but this attenuates as the cells continue to grow and mature in the constructs.

*JPH2* is a gene encoding an important protein for intracellular calcium release and contraction, and downregulation of *JPH2* has been implicated in patients with hypertrophic cardiomyopathy (37). The bioprinted constructs showed higher *JPH2* expression over time compared to the 2D cultures, again indicating further maturation of the cells in the bioprinted constructs. Expression of *SLN* is important for muscle relaxation, but overexpression of *SLN* can actually depress contractility in CMs (38). The *SLN* expression levels and video analysis support normal contraction of the bioprinted CMs.

*MYL2* encodes the myosin light chain 2 protein. This protein is involved in cardiac contractility and *MYL2* expression is used as a marker of the ventricular myocardium (39). The increased expression of *MYL2* in the bioprinted cells over time suggests these cells are becoming more mature and more ventricular in phenotype (40). A problem with iPSC-CMs is the heterogeneous CM population obtained after differentiation, with a mixture of atrial- and ventricular-like CMs. The increased *MYL2* levels is promising that the bioprinted constructs induce the iPSC-CMs to exhibit a more ventricular-like phenotype compared to 2D culture (4). The *KCNJ4* gene encodes a potassium channel protein, which is reportedly highly expressed in ventricular cardiomyocytes, again suggesting the bioprinted iPSC-CMs exhibit a more ventricular-like phenotype compared to 2D cultures (41, 42).

*MYH6* encodes myosin heavy chain 6 protein, which is associated with the formation of myosin and has known association with HLHS (3, 18). The 3D-bioprinted samples had much higher *MYH6* expression compared to the 2D samples, which suggests improved sarcomere function. *MHY7* is important for cardiac contraction. The higher expression of *MHY7* in the 3D-bioprinted constructs compared to the 2D cultures further validates the improved sarcomere function and contractility of these cells.

In previous work, we discovered that rare damaging variants in the *MYH6* gene, which encodes α-MHC, the major MHC of the developing human ventricle and adult atrium, are present in 10% of patients with HLHS and are found throughout the head, neck, and tail domains of the α-MHC (18). Furthermore, *MYH6* variants were also associated with reduced transplant-free survival in HLHS patients, indicating *MYH6* variants influence HLHS clinical outcome (18).

To elucidate mechanisms underlying *MYH6* variant pathology, we previously analysed patient-specific iPSCs from an HLHS family including a proband, an affected family member with coarctation of the aorta, another form of CHD, and a heart healthy family member to derive CMs from iPSCs. With this 2D iPSC-CM model of HLHS, we demonstrated that a *MYH6^R443P^* head domain variant exhibited delayed differentiation, sarcomere disorganization, compensatory expression of *MYH7*/β-MHC (the major MHC in adult ventricle) and reduced contractility (contracted more slowly, shortened less, and exhibited slower shortening and relaxation rates) (3).

We performed preliminary experiments using tissue constructs that were 3D-bioprinted using a mixture of these *MYH6* variant-carrying iPSC-CMs and wildtype iPSC-CMs. The experiment revealed dramatic differences in sarcomere length and organization, contractility, and gene expression of bioprinted constructs containing the variant cells compared to wildtype constructs (data not shown). These results are in line with our previous findings using 2D culture of iPSC-CMs containing an *MHY6* variant (3). This provides initial evidence that our tissue model successfully captured the disease phenotype. We are now positioned to generate 3D-bioprinted constructs made from variant iPSC-CMs alone and a mixture of both healthy and variant iPSC-CMs to understand phenotypic differences in a 3D microenvironment where cells are more mature and physiologically accurate.

A limitation of the present study is the focus on our experience with 3D-bioprinting wildtype iPSC-CMs. These cells are derived from biospecimens collected from healthy family members of patients with CHD. These cells are processed and stored in a clinical-grade tissue bank in a manner identical to the *MYH6* variant-carrying cells to allow for comparison between cells from a similar genetic background. It was necessary to use wildtype cells while developing our 3D-bioprinting and analysis protocols because their healthy and high-quality sarcomeres allowed us to develop protocols to quantitatively measure sarcomere quality and maturation in 3D tissue constructs.

While other groups have reported protocols for 3D-bioprinting iPSC-CMs (26, 28, 43–48), our methodology was specifically developed for iPSC-CMs derived from biospecimens stored in a clinical-grade tissue bank and obtained from families harbouring genetic variants causing CHD. We identified a commercial quality bioink, crosslinking strategy, and cell density that allows for consistent 3D-bioprinting of these clinically valuable cells. We also describe software and image analysis tools that allow for quantitative measurement of the quality and maturation of sarcomeres in a 3D tissue construct. Our methodology will enable clinically impactful research using patient-derived cells to study CHD and other cardiovascular diseases.

Another limitation of the present study is the relatively low sample number due to the large number of cells required for each print. The bioprinting protocol requires 5.0 × 10^7^–1.5 × 10^8^ cells per print to generate approximately 20 constructs. Future work will need to examine scaling to an appropriate cell number to create tissue volumes useful for surgical reconstruction. We may need to adjust the CM density in the constructs since the myocardium of human adults contains 2–4 × 10^9^ aligned CMs (49). We also limited the culture time to a maximum of 30 days after printing, and future studies will need to explore longer time points to understand long-term effects of the *MYH6* variant.

Future work will further characterize the 3D-bioprinted constructs and allow us to study the etiology of CHD. We will also use the tissue model to study candidate therapies for CHD. There is growing clinical interest in drugs that target myosin and improve the quality of sarcomeres in heart failure patients. Our model will allow the research community to study the effects of these and other drugs on the quality of sarcomeres. Our iPSC-CMs demonstrated rapid maturation after 3D-bioprinting, including formation of visible sarcomeres, which enables the efficient study of disease-associated variants in sarcomeric proteins. Importantly, this model enables the evaluation of patient-specific therapies.

Bioengineered, functional cardiac tissue also has exciting implications for surgical reconstruction and regeneration in patients with CHD. Such an approach does not currently exist and options for surgical reconstruction of congenitally dysfunctional ventricles are limited. 3D-bioprinting can be used in the development of living tissue grafts from autologous cell sources. These tissues can be cured of their genetic defects to restore their proper contractility. These functional tissues can then be used to surgically reconstruct congenitally abnormal hearts with the goal of restoring native activity and preventing the need for future surgeries.

We have developed a reliable method to 3D-bioprint patient-derived iPSC-CMs into 3D tissue constructs. Our results indicate the benefit of culturing these cells in 3D tissue constructs compared to 2D culture as evident through improved signs of CM maturity using analysis of sarcomere length and organization, contractility, and gene expression. This indicates our model is suitable for studying CHD etiology and potential treatments.

## Data Availability

The original contributions presented in the study are included in the article/**Supplementary Material**, further inquiries can be directed to the corresponding author.

## Supplementary material

Supplementary Video S1 Macroscopic video of contracting construct with wildtype cells.

Supplementary Video S2 Microscopic video of contracting construct with wildtype cells recorded at 40x.

Supplementary File S1 G-code commands for 3D-bioprinting.
