# Supplementary material for: 3D-bioprinting of patient-derived cardiac tissue models for studying congenital heart disease
Source: Front Cardiovasc Med. 2023 May 24;10:1162731. doi: 10.3389/fcvm.2023.1162731 (PMC10247285; doi:10.3389/fcvm.2023.1162731)
Supplement: Supplementary File S1 — G-code commands for 3D-bioprinting. [file Datasheet1.docx]

;Code for CellInk Inkredible Bioprinter

;Generated using CellInk's Heartware Slicer Software and Edited by Jayne Wolfe

;Code to print 4x4x0.6mm square in 35mm dish with UV curing at the end

;Total of 4 plates

;Pauses 15s to change out the plate

;Prints at 25C

M104 S25 ; set temperature

M109 S25 ; wait for temperature to be reached

G21 ; set units to millimeters

G90 ; use absolute coordinates

M83 ; use relative distances for extrusion

;Plate 1

;Layer 1

G1 Z0.200 F1800 ; 1st layer height 0.20mm

G1 X2.336 Y2.336 F1800 ; Linear move to start position

M760 ;Opens valve for printhead 1

G4 P0 ;Pause

G1 X1.168 Y2.336 E1 ;Begin extruding

G1 X0.0 Y2.336 E1

G1 X-1.1679999999999997 Y2.336 E1

G1 X-2.336 Y2.336 E0.03398 F1800.000

G1 X-2.336 Y1.168 E1

G1 X-2.336 Y0.0 E1

G1 X-2.336 Y-1.1679999999999997 E1

G1 X-2.336 Y-2.336 E0.03398

G1 X-1.168 Y-2.336 E1

G1 X0.0 Y-2.336 E1

G1 X1.1679999999999997 Y-2.336 E1

G1 X2.336 Y-2.336 E0.03398

G1 X2.336 Y-1.1829999999999998 E1

G1 X2.336 Y-0.029999999999999805 E1

G1 X2.336 Y1.1230000000000002 E1

G1 X2.336 Y2.276 E0.03354

M761

G1 X2.593 Y2.593 F1800

M760

G4 P0

G1 X1.2965 Y2.593 E1

G1 X0.0 Y2.593 E1

G1 X-1.2965 Y2.593 E1

G1 X-2.593 Y2.593 E0.03772 F1800.000

G1 X-2.593 Y1.2965 E1

G1 X-2.593 Y0.0 E1

G1 X-2.593 Y-1.2965 E1

G1 X-2.593 Y-2.593 E0.03772

G1 X-1.2965 Y-2.593 E1

G1 X0.0 Y-2.593 E1

G1 X1.2965 Y-2.593 E1

G1 X2.593 Y-2.593 E0.03772

G1 X2.593 Y-1.3115 E1

G1 X2.593 Y-0.03000000000000025 E1

G1 X2.593 Y1.2514999999999996 E1

G1 X2.593 Y2.533 E0.03728

M761

G1 X2.850 Y2.850 F3000

M760

G4 P0

G1 X1.425 Y2.85 E1

G1 X0.0 Y2.85 E1

G1 X-1.4250000000000003 Y2.85 E1

G1 X-2.850 Y2.850 E0.04146 F1800.000

G1 X-2.85 Y1.425 E1

G1 X-2.85 Y0.0 E1

G1 X-2.85 Y-1.4250000000000003 E1

G1 X-2.850 Y-2.850 E0.04146

G1 X-1.425 Y-2.85 E1

G1 X0.0 Y-2.85 E1

G1 X1.4250000000000003 Y-2.85 E1

G1 X2.850 Y-2.850 E0.04146

G1 X2.85 Y-1.44 E1

G1 X2.85 Y-0.029999999999999805 E1

G1 X2.85 Y1.3800000000000003 E1

G1 X2.850 Y2.790 E0.04102

M761

G1 X2.504 Y2.650 F3000

G1 X2.117 Y2.194 F3000

M760

G4 P0

G1 X2.117 Y0.7313333333333334 E1

G1 X2.117 Y-0.7313333333333332 E1

G1 X2.117 Y-2.194 E0.03287 F1800.000

G1 X1.853 Y-2.194 E0.00198

G1 X1.853 Y-0.7313333333333334 E1

G1 X1.853 Y0.7313333333333332 E1

G1 X1.853 Y2.194 E0.03287

G1 X1.588 Y2.194 E0.00198

G1 X1.588 Y0.7313333333333334 E1

G1 X1.588 Y-0.7313333333333332 E1

G1 X1.588 Y-2.194 E0.03287

G1 X1.323 Y-2.194 E0.00198

G1 X1.323 Y-0.7313333333333334 E1

G1 X1.323 Y0.7313333333333332 E1

G1 X1.323 Y2.194 E0.03287

G1 X1.059 Y2.194 E0.00198

G1 X1.059 Y0.7313333333333334 E1

G1 X1.059 Y-0.7313333333333332 E1

G1 X1.059 Y-2.194 E0.03287

G1 X0.794 Y-2.194 E0.00198

G1 X0.794 Y-0.7313333333333334 E1

G1 X0.794 Y0.7313333333333332 E1

G1 X0.794 Y2.194 E0.03287

G1 X0.529 Y2.194 E0.00198

G1 X0.529 Y0.7313333333333334 E1

G1 X0.529 Y-0.7313333333333332 E1

G1 X0.529 Y-2.194 E0.03287

G1 X0.265 Y-2.194 E0.00198

G1 X0.265 Y-0.7313333333333334 E1

G1 X0.265 Y0.7313333333333332 E1

G1 X0.265 Y2.194 E0.03287

G1 X-0.000 Y2.194 E0.00198

G1 X0.0 Y0.7313333333333334 E1

G1 X0.0 Y-0.7313333333333332 E1

G1 X-0.000 Y-2.194 E0.03287

G1 X-0.265 Y-2.194 E0.00198

G1 X-0.265 Y-0.7313333333333334 E1

G1 X-0.265 Y0.7313333333333332 E1

G1 X-0.265 Y2.194 E0.03287

G1 X-0.529 Y2.194 E0.00198

G1 X-0.529 Y0.7313333333333334 E1

G1 X-0.529 Y-0.7313333333333332 E1

G1 X-0.529 Y-2.194 E0.03287

G1 X-0.794 Y-2.194 E0.00198

G1 X-0.794 Y-0.7313333333333334 E1

G1 X-0.794 Y0.7313333333333332 E1

G1 X-0.794 Y2.194 E0.03287

G1 X-1.059 Y2.194 E0.00198

G1 X-1.059 Y0.7313333333333334 E1

G1 X-1.059 Y-0.7313333333333332 E1

G1 X-1.059 Y-2.194 E0.03287

G1 X-1.323 Y-2.194 E0.00198

G1 X-1.323 Y-0.7313333333333334 E1

G1 X-1.323 Y0.7313333333333332 E1

G1 X-1.323 Y2.194 E0.03287

G1 X-1.588 Y2.194 E0.00198

G1 X-1.588 Y0.7313333333333334 E1

G1 X-1.588 Y-0.7313333333333332 E1

G1 X-1.588 Y-2.194 E0.03287

G1 X-1.853 Y-2.194 E0.00198

G1 X-1.853 Y-0.7313333333333334 E1

G1 X-1.853 Y0.7313333333333332 E1

G1 X-1.853 Y2.194 E0.03287

G1 X-2.117 Y2.194 E0.00198

G1 X-2.117 Y0.7313333333333334 E1

G1 X-2.117 Y-0.7313333333333332 E1

G1 X-2.117 Y-2.194 E0.03287

M761 ;Closes the valve for printhead 1

; Layer 2

G1 Z0.400 F3000 ; Layer 2 height 0.20mm

G1 X1.679 Y1.679 F3000

M760 ;Opens valve for printhead 1

G4 P0 ;Pause

G1 X1.0074 Y1.679 E1 ;Begin extruding

G1 X0.3358000000000001 Y1.679 E1

G1 X-0.3358000000000001 Y1.679 E1

G1 X-1.0073999999999999 Y1.679 E1

G1 X-1.679 Y1.679 E0.05970 F900.000

G1 X-1.679 Y1.0074 E1

G1 X-1.679 Y0.3358000000000001 E1

G1 X-1.679 Y-0.3358000000000001 E1

G1 X-1.679 Y-1.0073999999999999 E1

G1 X-1.679 Y-1.679 E0.05970

G1 X-1.0074 Y-1.679 E1

G1 X-0.3358000000000001 Y-1.679 E1

G1 X0.3358000000000001 Y-1.679 E1

G1 X1.0073999999999999 Y-1.679 E1

G1 X1.679 Y-1.679 E0.05970

G1 X1.679 Y-1.0194 E1

G1 X1.679 Y-0.3598000000000001 E1

G1 X1.679 Y0.29979999999999984 E1

G1 X1.679 Y0.9593999999999998 E1

G1 X1.679 Y1.619 E0.05863

M761

G1 X2.307 Y2.307 F3000

M760

G4 P0

G1 X1.6478571428571427 Y2.307 E1

G1 X0.9887142857142857 Y2.307 E1

G1 X0.3295714285714286 Y2.307 E1

G1 X-0.3295714285714286 Y2.307 E1

G1 X-0.9887142857142859 Y2.307 E1

G1 X-1.6478571428571427 Y2.307 E1

G1 X-2.307 Y2.307 E0.08204 F900.000

G1 X-2.307 Y1.6478571428571427 E1

G1 X-2.307 Y0.9887142857142857 E1

G1 X-2.307 Y0.3295714285714286 E1

G1 X-2.307 Y-0.3295714285714286 E1

G1 X-2.307 Y-0.9887142857142859 E1

G1 X-2.307 Y-1.6478571428571427 E1

G1 X-2.307 Y-2.307 E0.08204

G1 X-1.6478571428571427 Y-2.307 E1

G1 X-0.9887142857142857 Y-2.307 E1

G1 X-0.3295714285714286 Y-2.307 E1

G1 X0.3295714285714286 Y-2.307 E1

G1 X0.9887142857142859 Y-2.307 E1

G1 X1.6478571428571427 Y-2.307 E1

G1 X2.307 Y-2.307 E0.08204

G1 X2.307 Y-1.6564285714285714 E1

G1 X2.307 Y-1.0058571428571428 E1

G1 X2.307 Y-0.3552857142857142 E1

G1 X2.307 Y0.2952857142857144 E1

G1 X2.307 Y0.9458571428571427 E1

G1 X2.307 Y1.5964285714285715 E1

G1 X2.307 Y2.247 E0.08097

M761

G1 X2.800 Y2.800 F3000

M760

G4 P0

G1 X2.0999999999999996 Y2.8 E1

G1 X1.4 Y2.8 E1

G1 X0.7000000000000002 Y2.8 E1

G1 X0.0 Y2.8 E1

G1 X-0.7000000000000002 Y2.8 E1

G1 X-1.3999999999999995 Y2.8 E1

G1 X-2.0999999999999996 Y2.8 E1

G1 X-2.800 Y2.800 E0.05658 F900.000

G1 X-2.8 Y2.0999999999999996 E1

G1 X-2.8 Y1.4 E1

G1 X-2.8 Y0.7000000000000002 E1

G1 X-2.8 Y0.0 E1

G1 X-2.8 Y-0.7000000000000002 E1

G1 X-2.8 Y-1.3999999999999995 E1

G1 X-2.8 Y-2.0999999999999996 E1

G1 X-2.800 Y-2.800 E0.05658

G1 X-2.0999999999999996 Y-2.8 E1

G1 X-1.4 Y-2.8 E1

G1 X-0.7000000000000002 Y-2.8 E1

G1 X0.0 Y-2.8 E1

G1 X0.7000000000000002 Y-2.8 E1

G1 X1.3999999999999995 Y-2.8 E1

G1 X2.0999999999999996 Y-2.8 E1

G1 X2.800 Y-2.800 E0.05658

G1 X2.8 Y-2.1075 E1

G1 X2.8 Y-1.4149999999999998 E1

G1 X2.8 Y-0.7224999999999997 E1

G1 X2.8 Y-0.029999999999999805 E1

G1 X2.8 Y0.6625000000000001 E1

G1 X2.8 Y1.3550000000000004 E1

G1 X2.8 Y2.0475000000000003 E1

G1 X2.800 Y2.740 E0.05597

M761

G1 X2.454 Y2.600 F3000

G1 X-1.333 Y1.145 F3000

M760

G4 P0

G1 X0.0 Y1.145 E1

G1 X1.333 Y1.145 E0.05760 F1800.000

G1 X1.333 Y0.382 E0.01648

G1 X0.0 Y0.382 E1

G1 X-1.333 Y0.382 E0.05760

G1 X-1.333 Y-0.382 E0.01648

G1 X0.0 Y-0.382 E1

G1 X1.333 Y-0.382 E0.05760

G1 X1.333 Y-1.145 E0.01648

G1 X0.0 Y-1.145 E1

G1 X-1.333 Y-1.145 E0.05760

M761 ;Closes the valve for printhead 1

; Layer 3

G1 Z0.600 F3000 ; Layer 3 height 0.20mm

G1 X0.0 Y-1.145 E1

G1 E-2.00000 F2400.00000

G1 X1.679 Y1.679 F3000

M760 ;Opens the valve for printhead 1

G4 P0 ;Pause

G1 X-0.32899999999999996 Y-0.20366666666666677 E1 ;Begin extruding

G1 X0.675 Y0.7376666666666665 E1

G1 E2.00000 F2400.00000

G1 X1.0074 Y1.679 E1

G1 X0.3358000000000001 Y1.679 E1

G1 X-0.3358000000000001 Y1.679 E1

G1 X-1.0073999999999999 Y1.679 E1

G1 X-1.679 Y1.679 E0.05970 F900.000

G1 X-1.679 Y1.0074 E1

G1 X-1.679 Y0.3358000000000001 E1

G1 X-1.679 Y-0.3358000000000001 E1

G1 X-1.679 Y-1.0073999999999999 E1

G1 X-1.679 Y-1.679 E0.05970

G1 X-1.0074 Y-1.679 E1

G1 X-0.3358000000000001 Y-1.679 E1

G1 X0.3358000000000001 Y-1.679 E1

G1 X1.0073999999999999 Y-1.679 E1

G1 X1.679 Y-1.679 E0.05970

G1 X1.679 Y-1.0194 E1

G1 X1.679 Y-0.3598000000000001 E1

G1 X1.679 Y0.29979999999999984 E1

G1 X1.679 Y0.9593999999999998 E1

G1 X1.679 Y1.619 E0.05863

M761

G1 X2.307 Y2.307 F7800.000

M760

G4 P0

G1 X1.6478571428571427 Y2.307 E1

G1 X0.9887142857142857 Y2.307 E1

G1 X0.3295714285714286 Y2.307 E1

G1 X-0.3295714285714286 Y2.307 E1

G1 X-0.9887142857142859 Y2.307 E1

G1 X-1.6478571428571427 Y2.307 E1

G1 X-2.307 Y2.307 E0.08204 F900.000

G1 X-2.307 Y1.6478571428571427 E1

G1 X-2.307 Y0.9887142857142857 E1

G1 X-2.307 Y0.3295714285714286 E1

G1 X-2.307 Y-0.3295714285714286 E1

G1 X-2.307 Y-0.9887142857142859 E1

G1 X-2.307 Y-1.6478571428571427 E1

G1 X-2.307 Y-2.307 E0.08204

G1 X-1.6478571428571427 Y-2.307 E1

G1 X-0.9887142857142857 Y-2.307 E1

G1 X-0.3295714285714286 Y-2.307 E1

G1 X0.3295714285714286 Y-2.307 E1

G1 X0.9887142857142859 Y-2.307 E1

G1 X1.6478571428571427 Y-2.307 E1

G1 X2.307 Y-2.307 E0.08204

G1 X2.307 Y-1.6564285714285714 E1

G1 X2.307 Y-1.0058571428571428 E1

G1 X2.307 Y-0.3552857142857142 E1

G1 X2.307 Y0.2952857142857144 E1

G1 X2.307 Y0.9458571428571427 E1

G1 X2.307 Y1.5964285714285715 E1

G1 X2.307 Y2.247 E0.08097

M761

G1 X2.800 Y2.800 F3000

M760

G4 P0

G1 X2.0999999999999996 Y2.8 E1

G1 X1.4 Y2.8 E1

G1 X0.7000000000000002 Y2.8 E1

G1 X0.0 Y2.8 E1

G1 X-0.7000000000000002 Y2.8 E1

G1 X-1.3999999999999995 Y2.8 E1

G1 X-2.0999999999999996 Y2.8 E1

G1 X-2.800 Y2.800 E0.05658 F900.000

G1 X-2.8 Y2.0999999999999996 E1

G1 X-2.8 Y1.4 E1

G1 X-2.8 Y0.7000000000000002 E1

G1 X-2.8 Y0.0 E1

G1 X-2.8 Y-0.7000000000000002 E1

G1 X-2.8 Y-1.3999999999999995 E1

G1 X-2.8 Y-2.0999999999999996 E1

G1 X-2.800 Y-2.800 E0.05658

G1 X-2.0999999999999996 Y-2.8 E1

G1 X-1.4 Y-2.8 E1

G1 X-0.7000000000000002 Y-2.8 E1

G1 X0.0 Y-2.8 E1

G1 X0.7000000000000002 Y-2.8 E1

G1 X1.3999999999999995 Y-2.8 E1

G1 X2.0999999999999996 Y-2.8 E1

G1 X2.800 Y-2.800 E0.05658

G1 X2.8 Y-2.1075 E1

G1 X2.8 Y-1.4149999999999998 E1

G1 X2.8 Y-0.7224999999999997 E1

G1 X2.8 Y-0.029999999999999805 E1

G1 X2.8 Y0.6625000000000001 E1

G1 X2.8 Y1.3550000000000004 E1

G1 X2.8 Y2.0475000000000003 E1

G1 X2.800 Y2.740 E0.05597

M761

G1 X2.454 Y2.600 F1800.000

G1 E-2.00000 F2400.00000

G1 X1.145 Y1.333 F1800.000

M760

G4 P0

G1 E2.00000 F2400.00000

G1 X1.145 Y0.6665 E1

G1 X1.145 Y0.0 E1

G1 X1.145 Y-0.6664999999999999 E1

G1 X1.145 Y-1.333 E0.05760 F900.000

G1 X0.7635000000000001 Y-1.333 E1

G1 X0.382 Y-1.333 E0.01648

G1 X0.382 Y-0.6665 E1

G1 X0.382 Y0.0 E1

G1 X0.382 Y0.6664999999999999 E1

G1 X0.382 Y1.333 E0.05760

G1 X0.0 Y1.333 E1

G1 X-0.382 Y1.333 E0.01648

G1 X-0.382 Y0.6665 E1

G1 X-0.382 Y0.0 E1

G1 X-0.382 Y-0.6664999999999999 E1

G1 X-0.382 Y-1.333 E0.05760

G1 X-0.7635000000000001 Y-1.333 E1

G1 X-1.145 Y-1.333 E0.01648

G1 X-1.145 Y-0.6665 E1

G1 X-1.145 Y0.0 E1

G1 X-1.145 Y0.6664999999999999 E1

G1 X-1.145 Y1.333 E0.05760

M761 ;Closes the valve for Printhead 1

; UV

M753 ; Raise both PHs

M713 ; Switch to PH3 (UV)

G0 X0.0 Y0.0

M753 ; Raise both PHs

M764 ; Turn on UV

G4 P15000; pause for 15,000 ms or 15 s

M765 ; Turn off UV

M753 ; Raise both PHs

M731 ; Switch to PH1

G0 X0.0 Y0.0

G1 X0 Y0

G1 Z50

G4 P15000; pause for 15,000 ms or 15s and switch out the plates

;Plate 2

;Layer 1

G1 Z0.200 F1800 ; 1st layer height 0.20mm

G1 X2.336 Y2.336 F1800 ; Linear move to start position

M760 ;Opens valve for printhead 1

G4 P0 ;Pause

G1 X1.168 Y2.336 E1 ;Begin extruding

G1 X0.0 Y2.336 E1

G1 X-1.1679999999999997 Y2.336 E1

G1 X-2.336 Y2.336 E0.03398 F1800.000

G1 X-2.336 Y1.168 E1

G1 X-2.336 Y0.0 E1

G1 X-2.336 Y-1.1679999999999997 E1

G1 X-2.336 Y-2.336 E0.03398

G1 X-1.168 Y-2.336 E1

G1 X0.0 Y-2.336 E1

G1 X1.1679999999999997 Y-2.336 E1

G1 X2.336 Y-2.336 E0.03398

G1 X2.336 Y-1.1829999999999998 E1

G1 X2.336 Y-0.029999999999999805 E1

G1 X2.336 Y1.1230000000000002 E1

G1 X2.336 Y2.276 E0.03354

M761

G1 X2.593 Y2.593 F1800

M760

G4 P0

G1 X1.2965 Y2.593 E1

G1 X0.0 Y2.593 E1

G1 X-1.2965 Y2.593 E1

G1 X-2.593 Y2.593 E0.03772 F1800.000

G1 X-2.593 Y1.2965 E1

G1 X-2.593 Y0.0 E1

G1 X-2.593 Y-1.2965 E1

G1 X-2.593 Y-2.593 E0.03772

G1 X-1.2965 Y-2.593 E1

G1 X0.0 Y-2.593 E1

G1 X1.2965 Y-2.593 E1

G1 X2.593 Y-2.593 E0.03772

G1 X2.593 Y-1.3115 E1

G1 X2.593 Y-0.03000000000000025 E1

G1 X2.593 Y1.2514999999999996 E1

G1 X2.593 Y2.533 E0.03728

M761

G1 X2.850 Y2.850 F3000

M760

G4 P0

G1 X1.425 Y2.85 E1

G1 X0.0 Y2.85 E1

G1 X-1.4250000000000003 Y2.85 E1

G1 X-2.850 Y2.850 E0.04146 F1800.000

G1 X-2.85 Y1.425 E1

G1 X-2.85 Y0.0 E1

G1 X-2.85 Y-1.4250000000000003 E1

G1 X-2.850 Y-2.850 E0.04146

G1 X-1.425 Y-2.85 E1

G1 X0.0 Y-2.85 E1

G1 X1.4250000000000003 Y-2.85 E1

G1 X2.850 Y-2.850 E0.04146

G1 X2.85 Y-1.44 E1

G1 X2.85 Y-0.029999999999999805 E1

G1 X2.85 Y1.3800000000000003 E1

G1 X2.850 Y2.790 E0.04102

M761

G1 X2.504 Y2.650 F3000

G1 X2.117 Y2.194 F3000

M760

G4 P0

G1 X2.117 Y0.7313333333333334 E1

G1 X2.117 Y-0.7313333333333332 E1

G1 X2.117 Y-2.194 E0.03287 F1800.000

G1 X1.853 Y-2.194 E0.00198

G1 X1.853 Y-0.7313333333333334 E1

G1 X1.853 Y0.7313333333333332 E1

G1 X1.853 Y2.194 E0.03287

G1 X1.588 Y2.194 E0.00198

G1 X1.588 Y0.7313333333333334 E1

G1 X1.588 Y-0.7313333333333332 E1

G1 X1.588 Y-2.194 E0.03287

G1 X1.323 Y-2.194 E0.00198

G1 X1.323 Y-0.7313333333333334 E1

G1 X1.323 Y0.7313333333333332 E1

G1 X1.323 Y2.194 E0.03287

G1 X1.059 Y2.194 E0.00198

G1 X1.059 Y0.7313333333333334 E1

G1 X1.059 Y-0.7313333333333332 E1

G1 X1.059 Y-2.194 E0.03287

G1 X0.794 Y-2.194 E0.00198

G1 X0.794 Y-0.7313333333333334 E1

G1 X0.794 Y0.7313333333333332 E1

G1 X0.794 Y2.194 E0.03287

G1 X0.529 Y2.194 E0.00198

G1 X0.529 Y0.7313333333333334 E1

G1 X0.529 Y-0.7313333333333332 E1

G1 X0.529 Y-2.194 E0.03287

G1 X0.265 Y-2.194 E0.00198

G1 X0.265 Y-0.7313333333333334 E1

G1 X0.265 Y0.7313333333333332 E1

G1 X0.265 Y2.194 E0.03287

G1 X-0.000 Y2.194 E0.00198

G1 X0.0 Y0.7313333333333334 E1

G1 X0.0 Y-0.7313333333333332 E1

G1 X-0.000 Y-2.194 E0.03287

G1 X-0.265 Y-2.194 E0.00198

G1 X-0.265 Y-0.7313333333333334 E1

G1 X-0.265 Y0.7313333333333332 E1

G1 X-0.265 Y2.194 E0.03287

G1 X-0.529 Y2.194 E0.00198

G1 X-0.529 Y0.7313333333333334 E1

G1 X-0.529 Y-0.7313333333333332 E1

G1 X-0.529 Y-2.194 E0.03287

G1 X-0.794 Y-2.194 E0.00198

G1 X-0.794 Y-0.7313333333333334 E1

G1 X-0.794 Y0.7313333333333332 E1

G1 X-0.794 Y2.194 E0.03287

G1 X-1.059 Y2.194 E0.00198

G1 X-1.059 Y0.7313333333333334 E1

G1 X-1.059 Y-0.7313333333333332 E1

G1 X-1.059 Y-2.194 E0.03287

G1 X-1.323 Y-2.194 E0.00198

G1 X-1.323 Y-0.7313333333333334 E1

G1 X-1.323 Y0.7313333333333332 E1

G1 X-1.323 Y2.194 E0.03287

G1 X-1.588 Y2.194 E0.00198

G1 X-1.588 Y0.7313333333333334 E1

G1 X-1.588 Y-0.7313333333333332 E1

G1 X-1.588 Y-2.194 E0.03287

G1 X-1.853 Y-2.194 E0.00198

G1 X-1.853 Y-0.7313333333333334 E1

G1 X-1.853 Y0.7313333333333332 E1

G1 X-1.853 Y2.194 E0.03287

G1 X-2.117 Y2.194 E0.00198

G1 X-2.117 Y0.7313333333333334 E1

G1 X-2.117 Y-0.7313333333333332 E1

G1 X-2.117 Y-2.194 E0.03287

M761 ;Closes the valve for printhead 1

; Layer 2

G1 Z0.400 F3000 ; Layer 2 height 0.20mm

G1 X1.679 Y1.679 F3000

M760 ;Opens valve for printhead 1

G4 P0 ;Pause

G1 X1.0074 Y1.679 E1 ;Begin extruding

G1 X0.3358000000000001 Y1.679 E1

G1 X-0.3358000000000001 Y1.679 E1

G1 X-1.0073999999999999 Y1.679 E1

G1 X-1.679 Y1.679 E0.05970 F900.000

G1 X-1.679 Y1.0074 E1

G1 X-1.679 Y0.3358000000000001 E1

G1 X-1.679 Y-0.3358000000000001 E1

G1 X-1.679 Y-1.0073999999999999 E1

G1 X-1.679 Y-1.679 E0.05970

G1 X-1.0074 Y-1.679 E1

G1 X-0.3358000000000001 Y-1.679 E1

G1 X0.3358000000000001 Y-1.679 E1

G1 X1.0073999999999999 Y-1.679 E1

G1 X1.679 Y-1.679 E0.05970

G1 X1.679 Y-1.0194 E1

G1 X1.679 Y-0.3598000000000001 E1

G1 X1.679 Y0.29979999999999984 E1

G1 X1.679 Y0.9593999999999998 E1

G1 X1.679 Y1.619 E0.05863

M761

G1 X2.307 Y2.307 F3000

M760

G4 P0

G1 X1.6478571428571427 Y2.307 E1

G1 X0.9887142857142857 Y2.307 E1

G1 X0.3295714285714286 Y2.307 E1

G1 X-0.3295714285714286 Y2.307 E1

G1 X-0.9887142857142859 Y2.307 E1

G1 X-1.6478571428571427 Y2.307 E1

G1 X-2.307 Y2.307 E0.08204 F900.000

G1 X-2.307 Y1.6478571428571427 E1

G1 X-2.307 Y0.9887142857142857 E1

G1 X-2.307 Y0.3295714285714286 E1

G1 X-2.307 Y-0.3295714285714286 E1

G1 X-2.307 Y-0.9887142857142859 E1

G1 X-2.307 Y-1.6478571428571427 E1

G1 X-2.307 Y-2.307 E0.08204

G1 X-1.6478571428571427 Y-2.307 E1

G1 X-0.9887142857142857 Y-2.307 E1

G1 X-0.3295714285714286 Y-2.307 E1

G1 X0.3295714285714286 Y-2.307 E1

G1 X0.9887142857142859 Y-2.307 E1

G1 X1.6478571428571427 Y-2.307 E1

G1 X2.307 Y-2.307 E0.08204

G1 X2.307 Y-1.6564285714285714 E1

G1 X2.307 Y-1.0058571428571428 E1

G1 X2.307 Y-0.3552857142857142 E1

G1 X2.307 Y0.2952857142857144 E1

G1 X2.307 Y0.9458571428571427 E1

G1 X2.307 Y1.5964285714285715 E1

G1 X2.307 Y2.247 E0.08097

M761

G1 X2.800 Y2.800 F3000

M760

G4 P0

G1 X2.0999999999999996 Y2.8 E1

G1 X1.4 Y2.8 E1

G1 X0.7000000000000002 Y2.8 E1

G1 X0.0 Y2.8 E1

G1 X-0.7000000000000002 Y2.8 E1

G1 X-1.3999999999999995 Y2.8 E1

G1 X-2.0999999999999996 Y2.8 E1

G1 X-2.800 Y2.800 E0.05658 F900.000

G1 X-2.8 Y2.0999999999999996 E1

G1 X-2.8 Y1.4 E1

G1 X-2.8 Y0.7000000000000002 E1

G1 X-2.8 Y0.0 E1

G1 X-2.8 Y-0.7000000000000002 E1

G1 X-2.8 Y-1.3999999999999995 E1

G1 X-2.8 Y-2.0999999999999996 E1

G1 X-2.800 Y-2.800 E0.05658

G1 X-2.0999999999999996 Y-2.8 E1

G1 X-1.4 Y-2.8 E1

G1 X-0.7000000000000002 Y-2.8 E1

G1 X0.0 Y-2.8 E1

G1 X0.7000000000000002 Y-2.8 E1

G1 X1.3999999999999995 Y-2.8 E1

G1 X2.0999999999999996 Y-2.8 E1

G1 X2.800 Y-2.800 E0.05658

G1 X2.8 Y-2.1075 E1

G1 X2.8 Y-1.4149999999999998 E1

G1 X2.8 Y-0.7224999999999997 E1

G1 X2.8 Y-0.029999999999999805 E1

G1 X2.8 Y0.6625000000000001 E1

G1 X2.8 Y1.3550000000000004 E1

G1 X2.8 Y2.0475000000000003 E1

G1 X2.800 Y2.740 E0.05597

M761

G1 X2.454 Y2.600 F3000

G1 X-1.333 Y1.145 F3000

M760

G4 P0

G1 X0.0 Y1.145 E1

G1 X1.333 Y1.145 E0.05760 F1800.000

G1 X1.333 Y0.382 E0.01648

G1 X0.0 Y0.382 E1

G1 X-1.333 Y0.382 E0.05760

G1 X-1.333 Y-0.382 E0.01648

G1 X0.0 Y-0.382 E1

G1 X1.333 Y-0.382 E0.05760

G1 X1.333 Y-1.145 E0.01648

G1 X0.0 Y-1.145 E1

G1 X-1.333 Y-1.145 E0.05760

M761 ;Closes the valve for printhead 1

; Layer 3

G1 Z0.600 F3000 ; Layer 3 height 0.20mm

G1 X0.0 Y-1.145 E1

G1 E-2.00000 F2400.00000

G1 X1.679 Y1.679 F3000

M760 ;Opens the valve for printhead 1

G4 P0 ;Pause

G1 X-0.32899999999999996 Y-0.20366666666666677 E1 ;Begin extruding

G1 X0.675 Y0.7376666666666665 E1

G1 E2.00000 F2400.00000

G1 X1.0074 Y1.679 E1

G1 X0.3358000000000001 Y1.679 E1

G1 X-0.3358000000000001 Y1.679 E1

G1 X-1.0073999999999999 Y1.679 E1

G1 X-1.679 Y1.679 E0.05970 F900.000

G1 X-1.679 Y1.0074 E1

G1 X-1.679 Y0.3358000000000001 E1

G1 X-1.679 Y-0.3358000000000001 E1

G1 X-1.679 Y-1.0073999999999999 E1

G1 X-1.679 Y-1.679 E0.05970

G1 X-1.0074 Y-1.679 E1

G1 X-0.3358000000000001 Y-1.679 E1

G1 X0.3358000000000001 Y-1.679 E1

G1 X1.0073999999999999 Y-1.679 E1

G1 X1.679 Y-1.679 E0.05970

G1 X1.679 Y-1.0194 E1

G1 X1.679 Y-0.3598000000000001 E1

G1 X1.679 Y0.29979999999999984 E1

G1 X1.679 Y0.9593999999999998 E1

G1 X1.679 Y1.619 E0.05863

M761

G1 X2.307 Y2.307 F7800.000

M760

G4 P0

G1 X1.6478571428571427 Y2.307 E1

G1 X0.9887142857142857 Y2.307 E1

G1 X0.3295714285714286 Y2.307 E1

G1 X-0.3295714285714286 Y2.307 E1

G1 X-0.9887142857142859 Y2.307 E1

G1 X-1.6478571428571427 Y2.307 E1

G1 X-2.307 Y2.307 E0.08204 F900.000

G1 X-2.307 Y1.6478571428571427 E1

G1 X-2.307 Y0.9887142857142857 E1

G1 X-2.307 Y0.3295714285714286 E1

G1 X-2.307 Y-0.3295714285714286 E1

G1 X-2.307 Y-0.9887142857142859 E1

G1 X-2.307 Y-1.6478571428571427 E1

G1 X-2.307 Y-2.307 E0.08204

G1 X-1.6478571428571427 Y-2.307 E1

G1 X-0.9887142857142857 Y-2.307 E1

G1 X-0.3295714285714286 Y-2.307 E1

G1 X0.3295714285714286 Y-2.307 E1

G1 X0.9887142857142859 Y-2.307 E1

G1 X1.6478571428571427 Y-2.307 E1

G1 X2.307 Y-2.307 E0.08204

G1 X2.307 Y-1.6564285714285714 E1

G1 X2.307 Y-1.0058571428571428 E1

G1 X2.307 Y-0.3552857142857142 E1

G1 X2.307 Y0.2952857142857144 E1

G1 X2.307 Y0.9458571428571427 E1

G1 X2.307 Y1.5964285714285715 E1

G1 X2.307 Y2.247 E0.08097

M761

G1 X2.800 Y2.800 F3000

M760

G4 P0

G1 X2.0999999999999996 Y2.8 E1

G1 X1.4 Y2.8 E1

G1 X0.7000000000000002 Y2.8 E1

G1 X0.0 Y2.8 E1

G1 X-0.7000000000000002 Y2.8 E1

G1 X-1.3999999999999995 Y2.8 E1

G1 X-2.0999999999999996 Y2.8 E1

G1 X-2.800 Y2.800 E0.05658 F900.000

G1 X-2.8 Y2.0999999999999996 E1

G1 X-2.8 Y1.4 E1

G1 X-2.8 Y0.7000000000000002 E1

G1 X-2.8 Y0.0 E1

G1 X-2.8 Y-0.7000000000000002 E1

G1 X-2.8 Y-1.3999999999999995 E1

G1 X-2.8 Y-2.0999999999999996 E1

G1 X-2.800 Y-2.800 E0.05658

G1 X-2.0999999999999996 Y-2.8 E1

G1 X-1.4 Y-2.8 E1

G1 X-0.7000000000000002 Y-2.8 E1

G1 X0.0 Y-2.8 E1

G1 X0.7000000000000002 Y-2.8 E1

G1 X1.3999999999999995 Y-2.8 E1

G1 X2.0999999999999996 Y-2.8 E1

G1 X2.800 Y-2.800 E0.05658

G1 X2.8 Y-2.1075 E1

G1 X2.8 Y-1.4149999999999998 E1

G1 X2.8 Y-0.7224999999999997 E1

G1 X2.8 Y-0.029999999999999805 E1

G1 X2.8 Y0.6625000000000001 E1

G1 X2.8 Y1.3550000000000004 E1

G1 X2.8 Y2.0475000000000003 E1

G1 X2.800 Y2.740 E0.05597

M761

G1 X2.454 Y2.600 F1800.000

G1 E-2.00000 F2400.00000

G1 X1.145 Y1.333 F1800.000

M760

G4 P0

G1 E2.00000 F2400.00000

G1 X1.145 Y0.6665 E1

G1 X1.145 Y0.0 E1

G1 X1.145 Y-0.6664999999999999 E1

G1 X1.145 Y-1.333 E0.05760 F900.000

G1 X0.7635000000000001 Y-1.333 E1

G1 X0.382 Y-1.333 E0.01648

G1 X0.382 Y-0.6665 E1

G1 X0.382 Y0.0 E1

G1 X0.382 Y0.6664999999999999 E1

G1 X0.382 Y1.333 E0.05760

G1 X0.0 Y1.333 E1

G1 X-0.382 Y1.333 E0.01648

G1 X-0.382 Y0.6665 E1

G1 X-0.382 Y0.0 E1

G1 X-0.382 Y-0.6664999999999999 E1

G1 X-0.382 Y-1.333 E0.05760

G1 X-0.7635000000000001 Y-1.333 E1

G1 X-1.145 Y-1.333 E0.01648

G1 X-1.145 Y-0.6665 E1

G1 X-1.145 Y0.0 E1

G1 X-1.145 Y0.6664999999999999 E1

G1 X-1.145 Y1.333 E0.05760

M761 ;Closes the valve for Printhead 1

; UV

M753 ; Raise both PHs

M713 ; Switch to PH3 (UV)

G0 X0.0 Y0.0

M753 ; Raise both PHs

M764 ; Turn on UV

G4 P15000; pause for 15,000 ms or 15 s

M765 ; Turn off UV

M753 ; Raise both PHs

M731 ; Switch to PH1

G0 X0.0 Y0.0

G1 X0 Y0

G1 Z50

G4 P15000; pause for 15,000 ms or 15s and switch out the plates

;Plate 3

;Layer 1

G1 Z0.200 F1800 ; 1st layer height 0.20mm

G1 X2.336 Y2.336 F1800 ; Linear move to start position

M760 ;Opens valve for printhead 1

G4 P0 ;Pause

G1 X1.168 Y2.336 E1 ;Begin extruding

G1 X0.0 Y2.336 E1

G1 X-1.1679999999999997 Y2.336 E1

G1 X-2.336 Y2.336 E0.03398 F1800.000

G1 X-2.336 Y1.168 E1

G1 X-2.336 Y0.0 E1

G1 X-2.336 Y-1.1679999999999997 E1

G1 X-2.336 Y-2.336 E0.03398

G1 X-1.168 Y-2.336 E1

G1 X0.0 Y-2.336 E1

G1 X1.1679999999999997 Y-2.336 E1

G1 X2.336 Y-2.336 E0.03398

G1 X2.336 Y-1.1829999999999998 E1

G1 X2.336 Y-0.029999999999999805 E1

G1 X2.336 Y1.1230000000000002 E1

G1 X2.336 Y2.276 E0.03354

M761

G1 X2.593 Y2.593 F1800

M760

G4 P0

G1 X1.2965 Y2.593 E1

G1 X0.0 Y2.593 E1

G1 X-1.2965 Y2.593 E1

G1 X-2.593 Y2.593 E0.03772 F1800.000

G1 X-2.593 Y1.2965 E1

G1 X-2.593 Y0.0 E1

G1 X-2.593 Y-1.2965 E1

G1 X-2.593 Y-2.593 E0.03772

G1 X-1.2965 Y-2.593 E1

G1 X0.0 Y-2.593 E1

G1 X1.2965 Y-2.593 E1

G1 X2.593 Y-2.593 E0.03772

G1 X2.593 Y-1.3115 E1

G1 X2.593 Y-0.03000000000000025 E1

G1 X2.593 Y1.2514999999999996 E1

G1 X2.593 Y2.533 E0.03728

M761

G1 X2.850 Y2.850 F3000

M760

G4 P0

G1 X1.425 Y2.85 E1

G1 X0.0 Y2.85 E1

G1 X-1.4250000000000003 Y2.85 E1

G1 X-2.850 Y2.850 E0.04146 F1800.000

G1 X-2.85 Y1.425 E1

G1 X-2.85 Y0.0 E1

G1 X-2.85 Y-1.4250000000000003 E1

G1 X-2.850 Y-2.850 E0.04146

G1 X-1.425 Y-2.85 E1

G1 X0.0 Y-2.85 E1

G1 X1.4250000000000003 Y-2.85 E1

G1 X2.850 Y-2.850 E0.04146

G1 X2.85 Y-1.44 E1

G1 X2.85 Y-0.029999999999999805 E1

G1 X2.85 Y1.3800000000000003 E1

G1 X2.850 Y2.790 E0.04102

M761

G1 X2.504 Y2.650 F3000

G1 X2.117 Y2.194 F3000

M760

G4 P0

G1 X2.117 Y0.7313333333333334 E1

G1 X2.117 Y-0.7313333333333332 E1

G1 X2.117 Y-2.194 E0.03287 F1800.000

G1 X1.853 Y-2.194 E0.00198

G1 X1.853 Y-0.7313333333333334 E1

G1 X1.853 Y0.7313333333333332 E1

G1 X1.853 Y2.194 E0.03287

G1 X1.588 Y2.194 E0.00198

G1 X1.588 Y0.7313333333333334 E1

G1 X1.588 Y-0.7313333333333332 E1

G1 X1.588 Y-2.194 E0.03287

G1 X1.323 Y-2.194 E0.00198

G1 X1.323 Y-0.7313333333333334 E1

G1 X1.323 Y0.7313333333333332 E1

G1 X1.323 Y2.194 E0.03287

G1 X1.059 Y2.194 E0.00198

G1 X1.059 Y0.7313333333333334 E1

G1 X1.059 Y-0.7313333333333332 E1

G1 X1.059 Y-2.194 E0.03287

G1 X0.794 Y-2.194 E0.00198

G1 X0.794 Y-0.7313333333333334 E1

G1 X0.794 Y0.7313333333333332 E1

G1 X0.794 Y2.194 E0.03287

G1 X0.529 Y2.194 E0.00198

G1 X0.529 Y0.7313333333333334 E1

G1 X0.529 Y-0.7313333333333332 E1

G1 X0.529 Y-2.194 E0.03287

G1 X0.265 Y-2.194 E0.00198

G1 X0.265 Y-0.7313333333333334 E1

G1 X0.265 Y0.7313333333333332 E1

G1 X0.265 Y2.194 E0.03287

G1 X-0.000 Y2.194 E0.00198

G1 X0.0 Y0.7313333333333334 E1

G1 X0.0 Y-0.7313333333333332 E1

G1 X-0.000 Y-2.194 E0.03287

G1 X-0.265 Y-2.194 E0.00198

G1 X-0.265 Y-0.7313333333333334 E1

G1 X-0.265 Y0.7313333333333332 E1

G1 X-0.265 Y2.194 E0.03287

G1 X-0.529 Y2.194 E0.00198

G1 X-0.529 Y0.7313333333333334 E1

G1 X-0.529 Y-0.7313333333333332 E1

G1 X-0.529 Y-2.194 E0.03287

G1 X-0.794 Y-2.194 E0.00198

G1 X-0.794 Y-0.7313333333333334 E1

G1 X-0.794 Y0.7313333333333332 E1

G1 X-0.794 Y2.194 E0.03287

G1 X-1.059 Y2.194 E0.00198

G1 X-1.059 Y0.7313333333333334 E1

G1 X-1.059 Y-0.7313333333333332 E1

G1 X-1.059 Y-2.194 E0.03287

G1 X-1.323 Y-2.194 E0.00198

G1 X-1.323 Y-0.7313333333333334 E1

G1 X-1.323 Y0.7313333333333332 E1

G1 X-1.323 Y2.194 E0.03287

G1 X-1.588 Y2.194 E0.00198

G1 X-1.588 Y0.7313333333333334 E1

G1 X-1.588 Y-0.7313333333333332 E1

G1 X-1.588 Y-2.194 E0.03287

G1 X-1.853 Y-2.194 E0.00198

G1 X-1.853 Y-0.7313333333333334 E1

G1 X-1.853 Y0.7313333333333332 E1

G1 X-1.853 Y2.194 E0.03287

G1 X-2.117 Y2.194 E0.00198

G1 X-2.117 Y0.7313333333333334 E1

G1 X-2.117 Y-0.7313333333333332 E1

G1 X-2.117 Y-2.194 E0.03287

M761 ;Closes the valve for printhead 1

; Layer 2

G1 Z0.400 F3000 ; Layer 2 height 0.20mm

G1 X1.679 Y1.679 F3000

M760 ;Opens valve for printhead 1

G4 P0 ;Pause

G1 X1.0074 Y1.679 E1 ;Begin extruding

G1 X0.3358000000000001 Y1.679 E1

G1 X-0.3358000000000001 Y1.679 E1

G1 X-1.0073999999999999 Y1.679 E1

G1 X-1.679 Y1.679 E0.05970 F900.000

G1 X-1.679 Y1.0074 E1

G1 X-1.679 Y0.3358000000000001 E1

G1 X-1.679 Y-0.3358000000000001 E1

G1 X-1.679 Y-1.0073999999999999 E1

G1 X-1.679 Y-1.679 E0.05970

G1 X-1.0074 Y-1.679 E1

G1 X-0.3358000000000001 Y-1.679 E1

G1 X0.3358000000000001 Y-1.679 E1

G1 X1.0073999999999999 Y-1.679 E1

G1 X1.679 Y-1.679 E0.05970

G1 X1.679 Y-1.0194 E1

G1 X1.679 Y-0.3598000000000001 E1

G1 X1.679 Y0.29979999999999984 E1

G1 X1.679 Y0.9593999999999998 E1

G1 X1.679 Y1.619 E0.05863

M761

G1 X2.307 Y2.307 F3000

M760

G4 P0

G1 X1.6478571428571427 Y2.307 E1

G1 X0.9887142857142857 Y2.307 E1

G1 X0.3295714285714286 Y2.307 E1

G1 X-0.3295714285714286 Y2.307 E1

G1 X-0.9887142857142859 Y2.307 E1

G1 X-1.6478571428571427 Y2.307 E1

G1 X-2.307 Y2.307 E0.08204 F900.000

G1 X-2.307 Y1.6478571428571427 E1

G1 X-2.307 Y0.9887142857142857 E1

G1 X-2.307 Y0.3295714285714286 E1

G1 X-2.307 Y-0.3295714285714286 E1

G1 X-2.307 Y-0.9887142857142859 E1

G1 X-2.307 Y-1.6478571428571427 E1

G1 X-2.307 Y-2.307 E0.08204

G1 X-1.6478571428571427 Y-2.307 E1

G1 X-0.9887142857142857 Y-2.307 E1

G1 X-0.3295714285714286 Y-2.307 E1

G1 X0.3295714285714286 Y-2.307 E1

G1 X0.9887142857142859 Y-2.307 E1

G1 X1.6478571428571427 Y-2.307 E1

G1 X2.307 Y-2.307 E0.08204

G1 X2.307 Y-1.6564285714285714 E1

G1 X2.307 Y-1.0058571428571428 E1

G1 X2.307 Y-0.3552857142857142 E1

G1 X2.307 Y0.2952857142857144 E1

G1 X2.307 Y0.9458571428571427 E1

G1 X2.307 Y1.5964285714285715 E1

G1 X2.307 Y2.247 E0.08097

M761

G1 X2.800 Y2.800 F3000

M760

G4 P0

G1 X2.0999999999999996 Y2.8 E1

G1 X1.4 Y2.8 E1

G1 X0.7000000000000002 Y2.8 E1

G1 X0.0 Y2.8 E1

G1 X-0.7000000000000002 Y2.8 E1

G1 X-1.3999999999999995 Y2.8 E1

G1 X-2.0999999999999996 Y2.8 E1

G1 X-2.800 Y2.800 E0.05658 F900.000

G1 X-2.8 Y2.0999999999999996 E1

G1 X-2.8 Y1.4 E1

G1 X-2.8 Y0.7000000000000002 E1

G1 X-2.8 Y0.0 E1

G1 X-2.8 Y-0.7000000000000002 E1

G1 X-2.8 Y-1.3999999999999995 E1

G1 X-2.8 Y-2.0999999999999996 E1

G1 X-2.800 Y-2.800 E0.05658

G1 X-2.0999999999999996 Y-2.8 E1

G1 X-1.4 Y-2.8 E1

G1 X-0.7000000000000002 Y-2.8 E1

G1 X0.0 Y-2.8 E1

G1 X0.7000000000000002 Y-2.8 E1

G1 X1.3999999999999995 Y-2.8 E1

G1 X2.0999999999999996 Y-2.8 E1

G1 X2.800 Y-2.800 E0.05658

G1 X2.8 Y-2.1075 E1

G1 X2.8 Y-1.4149999999999998 E1

G1 X2.8 Y-0.7224999999999997 E1

G1 X2.8 Y-0.029999999999999805 E1

G1 X2.8 Y0.6625000000000001 E1

G1 X2.8 Y1.3550000000000004 E1

G1 X2.8 Y2.0475000000000003 E1

G1 X2.800 Y2.740 E0.05597

M761

G1 X2.454 Y2.600 F3000

G1 X-1.333 Y1.145 F3000

M760

G4 P0

G1 X0.0 Y1.145 E1

G1 X1.333 Y1.145 E0.05760 F1800.000

G1 X1.333 Y0.382 E0.01648

G1 X0.0 Y0.382 E1

G1 X-1.333 Y0.382 E0.05760

G1 X-1.333 Y-0.382 E0.01648

G1 X0.0 Y-0.382 E1

G1 X1.333 Y-0.382 E0.05760

G1 X1.333 Y-1.145 E0.01648

G1 X0.0 Y-1.145 E1

G1 X-1.333 Y-1.145 E0.05760

M761 ;Closes the valve for printhead 1

; Layer 3

G1 Z0.600 F3000 ; Layer 3 height 0.20mm

G1 X0.0 Y-1.145 E1

G1 E-2.00000 F2400.00000

G1 X1.679 Y1.679 F3000

M760 ;Opens the valve for printhead 1

G4 P0 ;Pause

G1 X-0.32899999999999996 Y-0.20366666666666677 E1 ;Begin extruding

G1 X0.675 Y0.7376666666666665 E1

G1 E2.00000 F2400.00000

G1 X1.0074 Y1.679 E1

G1 X0.3358000000000001 Y1.679 E1

G1 X-0.3358000000000001 Y1.679 E1

G1 X-1.0073999999999999 Y1.679 E1

G1 X-1.679 Y1.679 E0.05970 F900.000

G1 X-1.679 Y1.0074 E1

G1 X-1.679 Y0.3358000000000001 E1

G1 X-1.679 Y-0.3358000000000001 E1

G1 X-1.679 Y-1.0073999999999999 E1

G1 X-1.679 Y-1.679 E0.05970

G1 X-1.0074 Y-1.679 E1

G1 X-0.3358000000000001 Y-1.679 E1

G1 X0.3358000000000001 Y-1.679 E1

G1 X1.0073999999999999 Y-1.679 E1

G1 X1.679 Y-1.679 E0.05970

G1 X1.679 Y-1.0194 E1

G1 X1.679 Y-0.3598000000000001 E1

G1 X1.679 Y0.29979999999999984 E1

G1 X1.679 Y0.9593999999999998 E1

G1 X1.679 Y1.619 E0.05863

M761

G1 X2.307 Y2.307 F7800.000

M760

G4 P0

G1 X1.6478571428571427 Y2.307 E1

G1 X0.9887142857142857 Y2.307 E1

G1 X0.3295714285714286 Y2.307 E1

G1 X-0.3295714285714286 Y2.307 E1

G1 X-0.9887142857142859 Y2.307 E1

G1 X-1.6478571428571427 Y2.307 E1

G1 X-2.307 Y2.307 E0.08204 F900.000

G1 X-2.307 Y1.6478571428571427 E1

G1 X-2.307 Y0.9887142857142857 E1

G1 X-2.307 Y0.3295714285714286 E1

G1 X-2.307 Y-0.3295714285714286 E1

G1 X-2.307 Y-0.9887142857142859 E1

G1 X-2.307 Y-1.6478571428571427 E1

G1 X-2.307 Y-2.307 E0.08204

G1 X-1.6478571428571427 Y-2.307 E1

G1 X-0.9887142857142857 Y-2.307 E1

G1 X-0.3295714285714286 Y-2.307 E1

G1 X0.3295714285714286 Y-2.307 E1

G1 X0.9887142857142859 Y-2.307 E1

G1 X1.6478571428571427 Y-2.307 E1

G1 X2.307 Y-2.307 E0.08204

G1 X2.307 Y-1.6564285714285714 E1

G1 X2.307 Y-1.0058571428571428 E1

G1 X2.307 Y-0.3552857142857142 E1

G1 X2.307 Y0.2952857142857144 E1

G1 X2.307 Y0.9458571428571427 E1

G1 X2.307 Y1.5964285714285715 E1

G1 X2.307 Y2.247 E0.08097

M761

G1 X2.800 Y2.800 F3000

M760

G4 P0

G1 X2.0999999999999996 Y2.8 E1

G1 X1.4 Y2.8 E1

G1 X0.7000000000000002 Y2.8 E1

G1 X0.0 Y2.8 E1

G1 X-0.7000000000000002 Y2.8 E1

G1 X-1.3999999999999995 Y2.8 E1

G1 X-2.0999999999999996 Y2.8 E1

G1 X-2.800 Y2.800 E0.05658 F900.000

G1 X-2.8 Y2.0999999999999996 E1

G1 X-2.8 Y1.4 E1

G1 X-2.8 Y0.7000000000000002 E1

G1 X-2.8 Y0.0 E1

G1 X-2.8 Y-0.7000000000000002 E1

G1 X-2.8 Y-1.3999999999999995 E1

G1 X-2.8 Y-2.0999999999999996 E1

G1 X-2.800 Y-2.800 E0.05658

G1 X-2.0999999999999996 Y-2.8 E1

G1 X-1.4 Y-2.8 E1

G1 X-0.7000000000000002 Y-2.8 E1

G1 X0.0 Y-2.8 E1

G1 X0.7000000000000002 Y-2.8 E1

G1 X1.3999999999999995 Y-2.8 E1

G1 X2.0999999999999996 Y-2.8 E1

G1 X2.800 Y-2.800 E0.05658

G1 X2.8 Y-2.1075 E1

G1 X2.8 Y-1.4149999999999998 E1

G1 X2.8 Y-0.7224999999999997 E1

G1 X2.8 Y-0.029999999999999805 E1

G1 X2.8 Y0.6625000000000001 E1

G1 X2.8 Y1.3550000000000004 E1

G1 X2.8 Y2.0475000000000003 E1

G1 X2.800 Y2.740 E0.05597

M761

G1 X2.454 Y2.600 F1800.000

G1 E-2.00000 F2400.00000

G1 X1.145 Y1.333 F1800.000

M760

G4 P0

G1 E2.00000 F2400.00000

G1 X1.145 Y0.6665 E1

G1 X1.145 Y0.0 E1

G1 X1.145 Y-0.6664999999999999 E1

G1 X1.145 Y-1.333 E0.05760 F900.000

G1 X0.7635000000000001 Y-1.333 E1

G1 X0.382 Y-1.333 E0.01648

G1 X0.382 Y-0.6665 E1

G1 X0.382 Y0.0 E1

G1 X0.382 Y0.6664999999999999 E1

G1 X0.382 Y1.333 E0.05760

G1 X0.0 Y1.333 E1

G1 X-0.382 Y1.333 E0.01648

G1 X-0.382 Y0.6665 E1

G1 X-0.382 Y0.0 E1

G1 X-0.382 Y-0.6664999999999999 E1

G1 X-0.382 Y-1.333 E0.05760

G1 X-0.7635000000000001 Y-1.333 E1

G1 X-1.145 Y-1.333 E0.01648

G1 X-1.145 Y-0.6665 E1

G1 X-1.145 Y0.0 E1

G1 X-1.145 Y0.6664999999999999 E1

G1 X-1.145 Y1.333 E0.05760

M761 ;Closes the valve for Printhead 1

; UV

M753 ; Raise both PHs

M713 ; Switch to PH3 (UV)

G0 X0.0 Y0.0

M753 ; Raise both PHs

M764 ; Turn on UV

G4 P15000; pause for 15,000 ms or 15 s

M765 ; Turn off UV

M753 ; Raise both PHs

M731 ; Switch to PH1

G0 X0.0 Y0.0

G1 X0 Y0

G1 Z50

G4 P15000; pause for 15,000 ms or 15s and switch out the plates

;Plate 4

;Layer 1

G1 Z0.200 F1800 ; 1st layer height 0.20mm

G1 X2.336 Y2.336 F1800 ; Linear move to start position

M760 ;Opens valve for printhead 1

G4 P0 ;Pause

G1 X1.168 Y2.336 E1 ;Begin extruding

G1 X0.0 Y2.336 E1

G1 X-1.1679999999999997 Y2.336 E1

G1 X-2.336 Y2.336 E0.03398 F1800.000

G1 X-2.336 Y1.168 E1

G1 X-2.336 Y0.0 E1

G1 X-2.336 Y-1.1679999999999997 E1

G1 X-2.336 Y-2.336 E0.03398

G1 X-1.168 Y-2.336 E1

G1 X0.0 Y-2.336 E1

G1 X1.1679999999999997 Y-2.336 E1

G1 X2.336 Y-2.336 E0.03398

G1 X2.336 Y-1.1829999999999998 E1

G1 X2.336 Y-0.029999999999999805 E1

G1 X2.336 Y1.1230000000000002 E1

G1 X2.336 Y2.276 E0.03354

M761

G1 X2.593 Y2.593 F1800

M760

G4 P0

G1 X1.2965 Y2.593 E1

G1 X0.0 Y2.593 E1

G1 X-1.2965 Y2.593 E1

G1 X-2.593 Y2.593 E0.03772 F1800.000

G1 X-2.593 Y1.2965 E1

G1 X-2.593 Y0.0 E1

G1 X-2.593 Y-1.2965 E1

G1 X-2.593 Y-2.593 E0.03772

G1 X-1.2965 Y-2.593 E1

G1 X0.0 Y-2.593 E1

G1 X1.2965 Y-2.593 E1

G1 X2.593 Y-2.593 E0.03772

G1 X2.593 Y-1.3115 E1

G1 X2.593 Y-0.03000000000000025 E1

G1 X2.593 Y1.2514999999999996 E1

G1 X2.593 Y2.533 E0.03728

M761

G1 X2.850 Y2.850 F3000

M760

G4 P0

G1 X1.425 Y2.85 E1

G1 X0.0 Y2.85 E1

G1 X-1.4250000000000003 Y2.85 E1

G1 X-2.850 Y2.850 E0.04146 F1800.000

G1 X-2.85 Y1.425 E1

G1 X-2.85 Y0.0 E1

G1 X-2.85 Y-1.4250000000000003 E1

G1 X-2.850 Y-2.850 E0.04146

G1 X-1.425 Y-2.85 E1

G1 X0.0 Y-2.85 E1

G1 X1.4250000000000003 Y-2.85 E1

G1 X2.850 Y-2.850 E0.04146

G1 X2.85 Y-1.44 E1

G1 X2.85 Y-0.029999999999999805 E1

G1 X2.85 Y1.3800000000000003 E1

G1 X2.850 Y2.790 E0.04102

M761

G1 X2.504 Y2.650 F3000

G1 X2.117 Y2.194 F3000

M760

G4 P0

G1 X2.117 Y0.7313333333333334 E1

G1 X2.117 Y-0.7313333333333332 E1

G1 X2.117 Y-2.194 E0.03287 F1800.000

G1 X1.853 Y-2.194 E0.00198

G1 X1.853 Y-0.7313333333333334 E1

G1 X1.853 Y0.7313333333333332 E1

G1 X1.853 Y2.194 E0.03287

G1 X1.588 Y2.194 E0.00198

G1 X1.588 Y0.7313333333333334 E1

G1 X1.588 Y-0.7313333333333332 E1

G1 X1.588 Y-2.194 E0.03287

G1 X1.323 Y-2.194 E0.00198

G1 X1.323 Y-0.7313333333333334 E1

G1 X1.323 Y0.7313333333333332 E1

G1 X1.323 Y2.194 E0.03287

G1 X1.059 Y2.194 E0.00198

G1 X1.059 Y0.7313333333333334 E1

G1 X1.059 Y-0.7313333333333332 E1

G1 X1.059 Y-2.194 E0.03287

G1 X0.794 Y-2.194 E0.00198

G1 X0.794 Y-0.7313333333333334 E1

G1 X0.794 Y0.7313333333333332 E1

G1 X0.794 Y2.194 E0.03287

G1 X0.529 Y2.194 E0.00198

G1 X0.529 Y0.7313333333333334 E1

G1 X0.529 Y-0.7313333333333332 E1

G1 X0.529 Y-2.194 E0.03287

G1 X0.265 Y-2.194 E0.00198

G1 X0.265 Y-0.7313333333333334 E1

G1 X0.265 Y0.7313333333333332 E1

G1 X0.265 Y2.194 E0.03287

G1 X-0.000 Y2.194 E0.00198

G1 X0.0 Y0.7313333333333334 E1

G1 X0.0 Y-0.7313333333333332 E1

G1 X-0.000 Y-2.194 E0.03287

G1 X-0.265 Y-2.194 E0.00198

G1 X-0.265 Y-0.7313333333333334 E1

G1 X-0.265 Y0.7313333333333332 E1

G1 X-0.265 Y2.194 E0.03287

G1 X-0.529 Y2.194 E0.00198

G1 X-0.529 Y0.7313333333333334 E1

G1 X-0.529 Y-0.7313333333333332 E1

G1 X-0.529 Y-2.194 E0.03287

G1 X-0.794 Y-2.194 E0.00198

G1 X-0.794 Y-0.7313333333333334 E1

G1 X-0.794 Y0.7313333333333332 E1

G1 X-0.794 Y2.194 E0.03287

G1 X-1.059 Y2.194 E0.00198

G1 X-1.059 Y0.7313333333333334 E1

G1 X-1.059 Y-0.7313333333333332 E1

G1 X-1.059 Y-2.194 E0.03287

G1 X-1.323 Y-2.194 E0.00198

G1 X-1.323 Y-0.7313333333333334 E1

G1 X-1.323 Y0.7313333333333332 E1

G1 X-1.323 Y2.194 E0.03287

G1 X-1.588 Y2.194 E0.00198

G1 X-1.588 Y0.7313333333333334 E1

G1 X-1.588 Y-0.7313333333333332 E1

G1 X-1.588 Y-2.194 E0.03287

G1 X-1.853 Y-2.194 E0.00198

G1 X-1.853 Y-0.7313333333333334 E1

G1 X-1.853 Y0.7313333333333332 E1

G1 X-1.853 Y2.194 E0.03287

G1 X-2.117 Y2.194 E0.00198

G1 X-2.117 Y0.7313333333333334 E1

G1 X-2.117 Y-0.7313333333333332 E1

G1 X-2.117 Y-2.194 E0.03287

M761 ;Closes the valve for printhead 1

; Layer 2

G1 Z0.400 F3000 ; Layer 2 height 0.20mm

G1 X1.679 Y1.679 F3000

M760 ;Opens valve for printhead 1

G4 P0 ;Pause

G1 X1.0074 Y1.679 E1 ;Begin extruding

G1 X0.3358000000000001 Y1.679 E1

G1 X-0.3358000000000001 Y1.679 E1

G1 X-1.0073999999999999 Y1.679 E1

G1 X-1.679 Y1.679 E0.05970 F900.000

G1 X-1.679 Y1.0074 E1

G1 X-1.679 Y0.3358000000000001 E1

G1 X-1.679 Y-0.3358000000000001 E1

G1 X-1.679 Y-1.0073999999999999 E1

G1 X-1.679 Y-1.679 E0.05970

G1 X-1.0074 Y-1.679 E1

G1 X-0.3358000000000001 Y-1.679 E1

G1 X0.3358000000000001 Y-1.679 E1

G1 X1.0073999999999999 Y-1.679 E1

G1 X1.679 Y-1.679 E0.05970

G1 X1.679 Y-1.0194 E1

G1 X1.679 Y-0.3598000000000001 E1

G1 X1.679 Y0.29979999999999984 E1

G1 X1.679 Y0.9593999999999998 E1

G1 X1.679 Y1.619 E0.05863

M761

G1 X2.307 Y2.307 F3000

M760

G4 P0

G1 X1.6478571428571427 Y2.307 E1

G1 X0.9887142857142857 Y2.307 E1

G1 X0.3295714285714286 Y2.307 E1

G1 X-0.3295714285714286 Y2.307 E1

G1 X-0.9887142857142859 Y2.307 E1

G1 X-1.6478571428571427 Y2.307 E1

G1 X-2.307 Y2.307 E0.08204 F900.000

G1 X-2.307 Y1.6478571428571427 E1

G1 X-2.307 Y0.9887142857142857 E1

G1 X-2.307 Y0.3295714285714286 E1

G1 X-2.307 Y-0.3295714285714286 E1

G1 X-2.307 Y-0.9887142857142859 E1

G1 X-2.307 Y-1.6478571428571427 E1

G1 X-2.307 Y-2.307 E0.08204

G1 X-1.6478571428571427 Y-2.307 E1

G1 X-0.9887142857142857 Y-2.307 E1

G1 X-0.3295714285714286 Y-2.307 E1

G1 X0.3295714285714286 Y-2.307 E1

G1 X0.9887142857142859 Y-2.307 E1

G1 X1.6478571428571427 Y-2.307 E1

G1 X2.307 Y-2.307 E0.08204

G1 X2.307 Y-1.6564285714285714 E1

G1 X2.307 Y-1.0058571428571428 E1

G1 X2.307 Y-0.3552857142857142 E1

G1 X2.307 Y0.2952857142857144 E1

G1 X2.307 Y0.9458571428571427 E1

G1 X2.307 Y1.5964285714285715 E1

G1 X2.307 Y2.247 E0.08097

M761

G1 X2.800 Y2.800 F3000

M760

G4 P0

G1 X2.0999999999999996 Y2.8 E1

G1 X1.4 Y2.8 E1

G1 X0.7000000000000002 Y2.8 E1

G1 X0.0 Y2.8 E1

G1 X-0.7000000000000002 Y2.8 E1

G1 X-1.3999999999999995 Y2.8 E1

G1 X-2.0999999999999996 Y2.8 E1

G1 X-2.800 Y2.800 E0.05658 F900.000

G1 X-2.8 Y2.0999999999999996 E1

G1 X-2.8 Y1.4 E1

G1 X-2.8 Y0.7000000000000002 E1

G1 X-2.8 Y0.0 E1

G1 X-2.8 Y-0.7000000000000002 E1

G1 X-2.8 Y-1.3999999999999995 E1

G1 X-2.8 Y-2.0999999999999996 E1

G1 X-2.800 Y-2.800 E0.05658

G1 X-2.0999999999999996 Y-2.8 E1

G1 X-1.4 Y-2.8 E1

G1 X-0.7000000000000002 Y-2.8 E1

G1 X0.0 Y-2.8 E1

G1 X0.7000000000000002 Y-2.8 E1

G1 X1.3999999999999995 Y-2.8 E1

G1 X2.0999999999999996 Y-2.8 E1

G1 X2.800 Y-2.800 E0.05658

G1 X2.8 Y-2.1075 E1

G1 X2.8 Y-1.4149999999999998 E1

G1 X2.8 Y-0.7224999999999997 E1

G1 X2.8 Y-0.029999999999999805 E1

G1 X2.8 Y0.6625000000000001 E1

G1 X2.8 Y1.3550000000000004 E1

G1 X2.8 Y2.0475000000000003 E1

G1 X2.800 Y2.740 E0.05597

M761

G1 X2.454 Y2.600 F3000

G1 X-1.333 Y1.145 F3000

M760

G4 P0

G1 X0.0 Y1.145 E1

G1 X1.333 Y1.145 E0.05760 F1800.000

G1 X1.333 Y0.382 E0.01648

G1 X0.0 Y0.382 E1

G1 X-1.333 Y0.382 E0.05760

G1 X-1.333 Y-0.382 E0.01648

G1 X0.0 Y-0.382 E1

G1 X1.333 Y-0.382 E0.05760

G1 X1.333 Y-1.145 E0.01648

G1 X0.0 Y-1.145 E1

G1 X-1.333 Y-1.145 E0.05760

M761 ;Closes the valve for printhead 1

; Layer 3

G1 Z0.600 F3000 ; Layer 3 height 0.20mm

G1 X0.0 Y-1.145 E1

G1 E-2.00000 F2400.00000

G1 X1.679 Y1.679 F3000

M760 ;Opens the valve for printhead 1

G4 P0 ;Pause

G1 X-0.32899999999999996 Y-0.20366666666666677 E1 ;Begin extruding

G1 X0.675 Y0.7376666666666665 E1

G1 E2.00000 F2400.00000

G1 X1.0074 Y1.679 E1

G1 X0.3358000000000001 Y1.679 E1

G1 X-0.3358000000000001 Y1.679 E1

G1 X-1.0073999999999999 Y1.679 E1

G1 X-1.679 Y1.679 E0.05970 F900.000

G1 X-1.679 Y1.0074 E1

G1 X-1.679 Y0.3358000000000001 E1

G1 X-1.679 Y-0.3358000000000001 E1

G1 X-1.679 Y-1.0073999999999999 E1

G1 X-1.679 Y-1.679 E0.05970

G1 X-1.0074 Y-1.679 E1

G1 X-0.3358000000000001 Y-1.679 E1

G1 X0.3358000000000001 Y-1.679 E1

G1 X1.0073999999999999 Y-1.679 E1

G1 X1.679 Y-1.679 E0.05970

G1 X1.679 Y-1.0194 E1

G1 X1.679 Y-0.3598000000000001 E1

G1 X1.679 Y0.29979999999999984 E1

G1 X1.679 Y0.9593999999999998 E1

G1 X1.679 Y1.619 E0.05863

M761

G1 X2.307 Y2.307 F7800.000

M760

G4 P0

G1 X1.6478571428571427 Y2.307 E1

G1 X0.9887142857142857 Y2.307 E1

G1 X0.3295714285714286 Y2.307 E1

G1 X-0.3295714285714286 Y2.307 E1

G1 X-0.9887142857142859 Y2.307 E1

G1 X-1.6478571428571427 Y2.307 E1

G1 X-2.307 Y2.307 E0.08204 F900.000

G1 X-2.307 Y1.6478571428571427 E1

G1 X-2.307 Y0.9887142857142857 E1

G1 X-2.307 Y0.3295714285714286 E1

G1 X-2.307 Y-0.3295714285714286 E1

G1 X-2.307 Y-0.9887142857142859 E1

G1 X-2.307 Y-1.6478571428571427 E1

G1 X-2.307 Y-2.307 E0.08204

G1 X-1.6478571428571427 Y-2.307 E1

G1 X-0.9887142857142857 Y-2.307 E1

G1 X-0.3295714285714286 Y-2.307 E1

G1 X0.3295714285714286 Y-2.307 E1

G1 X0.9887142857142859 Y-2.307 E1

G1 X1.6478571428571427 Y-2.307 E1

G1 X2.307 Y-2.307 E0.08204

G1 X2.307 Y-1.6564285714285714 E1

G1 X2.307 Y-1.0058571428571428 E1

G1 X2.307 Y-0.3552857142857142 E1

G1 X2.307 Y0.2952857142857144 E1

G1 X2.307 Y0.9458571428571427 E1

G1 X2.307 Y1.5964285714285715 E1

G1 X2.307 Y2.247 E0.08097

M761

G1 X2.800 Y2.800 F3000

M760

G4 P0

G1 X2.0999999999999996 Y2.8 E1

G1 X1.4 Y2.8 E1

G1 X0.7000000000000002 Y2.8 E1

G1 X0.0 Y2.8 E1

G1 X-0.7000000000000002 Y2.8 E1

G1 X-1.3999999999999995 Y2.8 E1

G1 X-2.0999999999999996 Y2.8 E1

G1 X-2.800 Y2.800 E0.05658 F900.000

G1 X-2.8 Y2.0999999999999996 E1

G1 X-2.8 Y1.4 E1

G1 X-2.8 Y0.7000000000000002 E1

G1 X-2.8 Y0.0 E1

G1 X-2.8 Y-0.7000000000000002 E1

G1 X-2.8 Y-1.3999999999999995 E1

G1 X-2.8 Y-2.0999999999999996 E1

G1 X-2.800 Y-2.800 E0.05658

G1 X-2.0999999999999996 Y-2.8 E1

G1 X-1.4 Y-2.8 E1

G1 X-0.7000000000000002 Y-2.8 E1

G1 X0.0 Y-2.8 E1

G1 X0.7000000000000002 Y-2.8 E1

G1 X1.3999999999999995 Y-2.8 E1

G1 X2.0999999999999996 Y-2.8 E1

G1 X2.800 Y-2.800 E0.05658

G1 X2.8 Y-2.1075 E1

G1 X2.8 Y-1.4149999999999998 E1

G1 X2.8 Y-0.7224999999999997 E1

G1 X2.8 Y-0.029999999999999805 E1

G1 X2.8 Y0.6625000000000001 E1

G1 X2.8 Y1.3550000000000004 E1

G1 X2.8 Y2.0475000000000003 E1

G1 X2.800 Y2.740 E0.05597

M761

G1 X2.454 Y2.600 F1800.000

G1 E-2.00000 F2400.00000

G1 X1.145 Y1.333 F1800.000

M760

G4 P0

G1 E2.00000 F2400.00000

G1 X1.145 Y0.6665 E1

G1 X1.145 Y0.0 E1

G1 X1.145 Y-0.6664999999999999 E1

G1 X1.145 Y-1.333 E0.05760 F900.000

G1 X0.7635000000000001 Y-1.333 E1

G1 X0.382 Y-1.333 E0.01648

G1 X0.382 Y-0.6665 E1

G1 X0.382 Y0.0 E1

G1 X0.382 Y0.6664999999999999 E1

G1 X0.382 Y1.333 E0.05760

G1 X0.0 Y1.333 E1

G1 X-0.382 Y1.333 E0.01648

G1 X-0.382 Y0.6665 E1

G1 X-0.382 Y0.0 E1

G1 X-0.382 Y-0.6664999999999999 E1

G1 X-0.382 Y-1.333 E0.05760

G1 X-0.7635000000000001 Y-1.333 E1

G1 X-1.145 Y-1.333 E0.01648

G1 X-1.145 Y-0.6665 E1

G1 X-1.145 Y0.0 E1

G1 X-1.145 Y0.6664999999999999 E1

G1 X-1.145 Y1.333 E0.05760

M761 ;Closes the valve for Printhead 1

; UV

M753 ; Raise both PHs

M713 ; Switch to PH3 (UV)

G0 X0.0 Y0.0

M753 ; Raise both PHs

M764 ; Turn on UV

G4 P15000; pause for 15,000 ms or 15 s

M765 ; Turn off UV

M753 ; Raise both PHs

M731 ; Switch to PH1

G0 X0.0 Y0.0

G1 X0 Y0

G1 Z50

G1 Z50

M84 ;disable motors

;END
